# Recent advances in high performance poly(lactide): from “green” plasticization to super-tough materials via (reactive) compounding

**DOI:** 10.3389/fchem.2013.00032

**Published:** 2013-12-17

**Authors:** Georgio Kfoury, Jean-Marie Raquez, Fatima Hassouna, Jérémy Odent, Valérie Toniazzo, David Ruch, Philippe Dubois

**Affiliations:** ^1^Department of Advanced Materials and Structures, Public Research Center Henri TudorHautcharage, Luxembourg; ^2^Laboratory of Polymeric and Composite Materials, UMONS Research Institute for Materials Science and Engineering, Center for Innovation and Research in Materials and Polymers, University of MonsMons, Belgium

**Keywords:** poly(lactide), (reactive) compounding, mechanical properties, impact resistance, toughening

## Abstract

Due to its origin from renewable resources, its biodegradability, and recently, its industrial implementation at low costs, poly(lactide) (PLA) is considered as one of the most promising ecological, bio-sourced and biodegradable plastic materials to potentially and increasingly replace traditional petroleum derived polymers in many commodity and engineering applications. Beside its relatively high rigidity [high tensile strength and modulus compared with many common thermoplastics such as poly(ethylene terephthalate) (PET), high impact poly(styrene) (HIPS) and poly(propylene) (PP)], PLA suffers from an inherent brittleness, which can limit its applications especially where mechanical toughness such as plastic deformation at high impact rates or elongation is required. Therefore, the curve plotting stiffness vs. impact resistance and ductility must be shifted to higher values for PLA-based materials, while being preferably fully bio-based and biodegradable upon the application. This review aims to establish a state of the art focused on the recent progresses and preferably economically viable strategies developed in the literature for significantly improve the mechanical performances of PLA. A particular attention is given to plasticization as well as to impact resistance modification of PLA in the case of (reactive) blending PLA-based systems.

## Introduction

**Figure 1:**
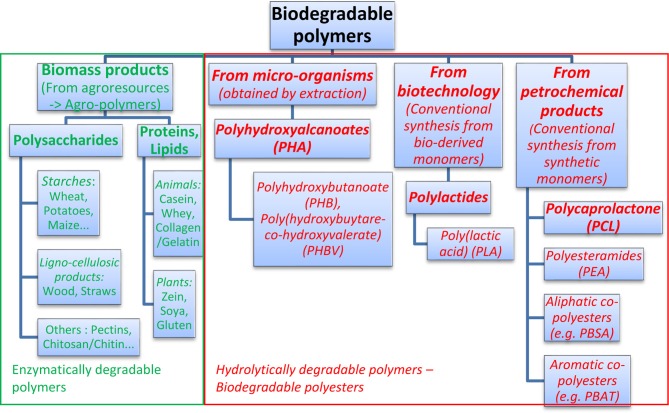
**Classification of the most known biodegradable polymers**.

Over the past decade, there has been a significant research interest on compostable and/or biodegradable polymers in order to alleviate solid waste disposal problems related with petro-based plastics (Lim et al., 2008). These biodegradable polymeric materials are increasingly used today in packaging, agricultural, medical, pharmaceutical, and other areas (Rabetafika et al., 2006; Vroman and Tighzert, 2009). Two main classes of biodegradable polymers can be distinguished (Vroman and Tighzert, 2009) (Figure 1):

*– Natural and synthetic biodegradable polymers* produced from feedstocks derived from biological or renewable resources available in large quantities;
*– Synthetic biodegradable polymers* produced from feedstocks derived from non-renewable petroleum resources.

Aliphatic polyesters represent a large part of biodegradable polymers. They are considered as hydrolytically degradable polymers due to the presence in their backbone of hydrolytically sensitive chemical bonds, that is, ester moieties (Li, 1999; Nair and Laurencin, 2007). There are two routes generally used to chemically develop biodegradable polyesters; step (condensation) polymerization and ring-opening polymerization (ROP) (Nair and Laurencin, 2007). Due to the absence of any by-products released during condensation process, ROP is thereby the most used pathway to prepare biodegradable polyesters. Among them, the most extensively investigated polymers are the poly(α-hydroxyacid)s, which include poly(glycolic acid) and the stereoisomeric copolymers of poly(lactic acid). Due to the commercial and low cost production of high molecular weight polymers using ROP, poly(lactide) (PLA) is one of the most studied candidates (Lim et al., 2008). Indeed, this polymer represents one of the stiffest organic materials with a Young's modulus of ca. 3 GPa, together with good optical and thermal properties [melting temperature (*T_m_*) of ca. 170°C and a glass transition temperature (*T_g_*) of ca. 60°C]. In addition, PLA is directly derived from renewable resources, making it environmentally sustainable in terms of depletion of petroleum resources and CO_2_-release. Due to these attributes, PLA holds tremendous promises as an alternative to the ubiquitous petroleum-based materials as shown in Table 1. For instance, compared with the general purpose polystyrene (GPPS), PLA has not only comparable tensile strength and modulus, but also exhibits very similar inherent brittleness (see Table 1). However, despite its numerous advantages such as good optical, physical, mechanical properties (high flexural and tensile moduli and strengths), the inherent brittleness significantly impedes its applications in many fields when a high level of mechanical strength is required.

**Table 1:** **PLA mechanical properties compared to those of most common polymers used in commodity applications [Copyright ©(2011) Wiley and Sons; used with permission from Liu and Zhang (2011)]**.

	**PLA**	**PET**	**PS**	**HIPS**	**PP**
*T_g_* (°C)	55–65	75	105	–	10
Tensile strength at break (MPa)	53	54	45	23	31
Tensile modulus (GPa)	3.4	2.8	2.9	2.1	0.9
Elongation at break (%)	6	130	7	45	120
Notched Izod impact strength (J/m)	13	59	27	123	27 (i-PP)
Gardner impact (J)	0.06	0.32	0.51	11.30	0.79
Cost ($/lb)^a^	1–1.5	0.70–0.72	0.99–1.01	1.01–1.03	1.15–1.17

*PET, Poly(ethylene terephthalate); PS, Polystyrene; HIPS, High-impact polystyrene; PP, Polypropylene; i-PP, Isotactic polypropylene homopolymer*.

a *Cost cited from “Plastic News”, March 31, 2011 except PLA resin*.

The mechanical resistance of a material is its ability to withstand the application of a sudden load without failure by dissipation of energy of the impact blow. There are two general failure modes, namely *brittle fracture* and *ductile fracture*. While *brittle fracture*, usually resulting of highly concentrated *crazing*, is characterized by a relatively low energy dissipation and a short nearly linear dependence of load–deformation before fracture, a *ductile fracture* is characterized by a high energy dissipation and a large-scale deformation (plastic yielding and plastic flow) (Bucknall, 1978; White, 1984; Argon and Cohen, 1990; Perkins, 1999). A *brittle-ductile transition* is accordingly defined as the point at which the fracture energy increases significantly with a mode of failure passing from brittle fracture to ductile fracture. The importance of this transition zone depends mainly on the strain nature and rate, the temperature gradient, and the specimen geometry (Perkins, 1999). For instance, the same material can exhibit higher brittleness at low temperatures and/or high testing speeds. Mechanical resistance of polymers may be evaluated in terms of the energy absorbed by the specimen during testing by various methods including (Pearson Raymond, 2000):

– *Tensile testing*: The area under the stress strain curve is often used to quantify toughness. However, different stress-strain curve shapes indicating different mechanical behaviors and responses to the impact loading may dissipate the same impact energy.
– *Impact testing*: The energy required to break the sample which is usually entailed by a hammer is measured. The related impact strength is expressed in terms of the difference between the potential energy of the striker before and after the impact. It is generally obtained by dividing the energy required to break the sample by the sample width or cross-sectional area. For impact testing, three different tests are typically performed such as Izod (ASTM D256 where samples are clamped as a cantilever), Charpy (ASTM D6110 unclamped samples are supported at both ends) and Dynstat (DIN 53453 where samples are unclamped at the lower end), which can be done in either a notched or un-notched state.
– Falling weight tests where a projectile propelled onto the specimen or dropped on it under the force of gravity is used to measure the impact energy. Gardner impact tester is a well-known example of this type of instrument which offers the advantage over impact testing method that the fracture shape can be also analyzed.

Typically, like conventional brittle thermoplastics, the reason for brittleness of PLA is strain and stress localizations at its use temperature, which is usually below its glass transition and brittle to ductile transition temperature. Under mechanical loading PLA deforms involving highly localized *crazing mechanism*. As at room temperature its yield stress is superior to the critical stress value for crack formation and propagation, catastrophic damage and break can most likely occur at low deformation and in the elastic zone. The strain-localization can be suppressed namely by compounding the brittle polymer with various softening and toughening agents including plasticizers and rubbery polymers or impact modifiers. However, the most preferred way is to blend PLA with rubbery polymers in order achieve a good toughness-stiffness balance without largely scarifying its glass transition temperature. Like many tough polymer blends, PLA blends can undergo one or a combination of the most known toughening mechanisms, namely *multiple crazing, shear yielding, cavitation and debonding* (Petchwattana et al., 2012). The mechanical energy is therefore transferred to the plastic flow and dissipated through a large volume fraction of material. The energy dissipation mechanisms retard or stop crack initiation and propagation through the polymer, and ultimately result in a material with improved toughness. There are several factors that can influence the amount of toughening, mainly related to the matrix polymer (Kramer, 1983), the rubber phase (type, particle size, concentration, strength and morphology), and the rubber-matrix interfacial interaction. For instance, the correlations between the deformation morphologies (mainly under tensile and impact testing) and the resulting mechanical properties reveal that the blend compatibility and related morphologies are important factors to influence the toughening mechanisms. The toughening mechanisms can be analyzed through several aspects, including stress whitening, matrix ligament thickness, microstructure evolution under testing, and morphology features of the fracture surface of the impacted sample. For instance, when the matrix ligament thickness is below the critical value, the blends deform to a large extent because of shear yield initiated by stress concentrations and interfacial de-bonding. This may result in the formation of fibers in both tensile and impact samples and the dissipation of a large amount of energy (Han and Huang, 2011).

Many strategies, namely the incorporation of a variety of soft polymers or rubbers, addition of rigid fillers and fibers, and modification of crystalline morphology, have been developed in the literature during the last decades in order to enhance the general toughness of PLA, while maintaining its stiffness-toughness balance acceptable (Anderson et al., 2008; Liu and Zhang, 2011). An optimal toughness balance can be obtained with 10–30% of toughening agents, even if little improvement can be seen by the addition of 5–10% of the latter (Mascia and Xanthos, 1992; Anderson et al., 2008; Liu and Zhang, 2011). In this regard, blending represents an economically viable approach such as plasticization, (reactive) compounding with a variety of flexible/soft polymers or rubbers and the addition of rigid fillers. In this report, an update on the strategies recently developed in the literature to significantly and effectively improve PLA's mechanical properties, will be discussed on its toughening and impact resistance properties.

## Approaches for the improvement of PLA's mechanical properties by modifying its inherent crystalline structure

The impact strength of semicrystalline polymers usually varies inversely with the percent crystallinity (Mercier et al., 1965). It is likely that crystallites act as stress concentrators, causing the stress acting on a small volume of the material to be much greater than the average stress applied to the whole sample. As a result, the material breaks at a stress that is less than the expected critical value. Also, crystallites are seen to reduce multiple crazing and shear yielding (Pecorini and Hertzberg, 1993), both energy-dissipative mechanisms of polymer matrices. The size and number of these crystalline structures have a profound influence on impact resistance. It is generally agreed that impact resistance and the brittle to ductile transition temperature are inversely related to spherulite size and morphology which can be tuned by controlling the cooling and drawing rates via thermal and mechanical treatments, respectively (Hammer et al., 1959; Ohlberg et al., 1959; Barish, 1962). This part of the study concerns the PLA matrix itself. In this regard we will report the main approaches that tune up the relationships “physical treatments—crystalline structure—mechanical properties” in order to improve the mechanical properties of PLA-based materials.

### Thermal treatments—annealing

The effect of annealing treatment on thermal, mechanical and fracture behavior of PLA was investigated. Most of the studies demonstrated that the increase of PLA crystallinity usually leads to; an improvement of its overall mechanical and heat resistance behaviors (Perego et al., 1996; Park et al., 2006; Yu et al., 2008; Nascimento et al., 2010). For instance, (Perego et al., 1996) evidenced that annealed PLA possess higher heat resistance, elastic moduli (tensional and flexural), Izod impact strength. Park et al. (2006) and Nascimento et al. (2010). Annealed PLA under different conditions to obtain several microstructures with varying spherulite size and density. They demonstrated that heat resistance is dramatically improved as crystallinity. Furthermore, the quasi-static fracture toughness of PLA decreases with increase of crystallinity corresponding to decrease of amorphous region; on the other hand, the impact fracture toughness tends to increase with crystallinity. The crack growth behaviors of the PLA specimens having different crystallinity exhibited that under quasi-static loading, disappearance of multiple crazes in the crack-tip region results in the decrease of the fracture toughness with crystallinity. On the contrary, under impact loading, the increase of the fracture toughness with crystallinity is considered to be related to the increase of fibril formation. Finally, for the amorphous PLA, the static toughness was higher than the impact one; mainly owing to extensive multiple craze formation at the static rate. On the contrary, for the crystallized PLA, the impact toughness became larger than the static one due to formation of fibril structure at the impact rate (Gamez-Perez, 2010). Gamez-Perez (2010) applied annealing treatment on two commercial grades of PLA from NatureWorks® (2002D and 4032D) of comparable average molecular weights (*M_w_*) of 212 and 207 kDa, respectively, but they exhibited different optical purities, that is, d-lactic monomer contents of 4.25 and 2%, respectively (Natureworks®, 2005, 2006; Li and Huneault, 2007; Xiao et al., 2009; Carrasco et al., 2010). Annealing the sheets was performed using an oven at 60°C for 20 min, followed by a rapid quenching. The nomenclature employed was “PLA-X” and “PLA-XT” for extruded and thermally treated films, respectively. “X” is set as 96 and 98 for PLAs for a content of 95.75 and 98% l-lactic monomer, respectively. From Table 2, it results that the heating at temperatures close to the glass transition temperature (*T_g_*) with the subsequent quenching treatment produces a “de-aging effect,” with an increase of the free-volume of polymeric chains, as highlighted by the decrease of the *T_g_*. The increase in the system potential energy was also shown by the disappearance of the endothermic peak at *T_g_*. As a consequence, annealing promotes a brittle-to-ductile change in the fracture behavior of PLA with a decrease of the tensile strength and stiffness and yield stress, regardless the d-lactic isomer content. A shear yielding with a localized neck formation thereby appeared. The fracture parameters, assessed by the EWF method used to characterize the fracture toughness of PLA showed a great enhancement of the toughness after the annealing and quenching treatments. Regarding the influence of the D-lactic isomer content in PLA films, when they were in a glassy stage, no remarkable differences were noticed out in the mechanical properties and fracture behavior. Only when the films were in a de-aged form, the differences in the stiffness of both PLA grades had been revealed. The optical purity, the elastic modulus and the tensile strength were high. However, the deformation to break was still low, only passing from 17% (PLA-98) to 24% (PLA-96).

**Table 2:** **Effect of some (thermo)mechanical treatments and processing on the thermal and mechanical properties of PLA**.

**Material**	***T_g_*(°C)**	***T_m_*(°C)**	**Δ*H_cc_*(J/g)**	**Δ*H_m_*(J/g)**	***X_c_*(%)**	**Yield stress σ *_y_* (MPa)**	**Young's modulus E (GPa)**	**Elongation at break ε*_b_* (%)**	**Charpy (KJ/m^2^)**	**Izod impact (KJ/m^2^)**	**References**
PLA-96	60	148	–	1	1	56.2 ± 0.7	4.0 ± 0.2	24 ± 5			Gamez-Perez, 2010
PLA-98	61	164	29	31	2	58.4 ± 0.5	4.3 ± 0.1	17 ± 4			
PLA-96T	56	148	–	1	1	47.3 ± 1.1	3.3 ± 0.2	456 ± 100			
PLA-98T	57	165	31	34	3	53.4 ± 0.6	3.5 ± 0.3	422 ± 50			
Un-oriented						47.0	3.65	1.5	12.5	1.6	Grijpma et al., 2002
Oriented (λ = 2.5)						73.3	4.49	48.2	35.9	5.9	
Oriented (λ = 3.4)						66.3	3.74	21.8	No break	52.0	
PLA-I						65.6 ± 1.3	3.7 ± 0.1	4.0 ± 0.8			Carrasco et al., 2010
PLA-EI						65.2 ± 0.9	3.9 ± 0.1	5.4 ± 0.6			
PLA-IA						75.4 ± 0.9	4.1 ± 0.1	2.5 ± 0.2			
PLA-EIA						77.0 ± 1.1	4.1 ± 0.1	3.3 ± 0.3			

*I, Injected; IA, Injected then Annealed; EI, Extruded then Annealed; EIA, Extruded then Injected then Annealed*.

### Thermomechanical treatments—self-reinforcing polymeric materials procedures (SRPMs)—alignment and orientation procedures

Although polymeric composites are referred to as multi-phase or hetero-composites, self-reinforced polymeric materials (SRPMs) are referred to as single-phase or homo-composites because the same polymer forms both the reinforcing and the matrix phases. The basic concept of self-reinforcement is to create a one-, two- or three-dimensional alignment (1D, 2D, or 3D alignment, respectively) within the matrix to fulfill the role of matrix reinforcement. As a result, the generated structure has to possess a higher stiffness and strength than the matrix as well as to be “well-bonded” to the matrix polymer. Consequently, the stress can be transferred from the “weak” matrix to the “strong” reinforcing structure, according to the “working principle” of all composites. The reinforcing structure can be produced during one (*in situ*) or more processing steps (*ex situ*) (Kmetty et al., 2010). A driving force for SRPMs is the possibility of manufacturing lightweight parts and structures because the density of SRPMs is well-below those of traditional filled polymers, where the “heavier” reinforcements incorporated in the polymeric matrix are of, e.g., glass fibers (density: 2.5–2.9 g.cm^−3^), carbon fibers (density:1.7–1.9 g.cm^−3^), basalt fibers (density: 2.7–3.0 g.cm^−3^), aramid fibers (density: 1.38–1.44 g.cm^−3^) and/or fillers like talc (density: 2.7–2.8 g.cm^−3^), chalk (density: 1.1–2.5 g.cm^−3^) and silica (density: 2.1–2.6 g.cm^−3^) (Kmetty et al., 2010). Furthermore, the ease of recycling SRPMs must be emphasized when reprocessing via re-melting is targeted. The concepts used to produce SRPMs can be also adapted to biodegradable polymers for improving their property profiles. Reinforcing a PLA matrix by embedding PLA fibers enables to respond the demands for high strength and stiffness required for many applications. The development of high-stiffness and high-strength polymeric fibers is essential to imparting superior mechanical properties for the resulting PLA SRCs (Matabola et al., 2009). The mechanical properties of fibers can be increased via molecular orientation during spinning and drawing (Alcock et al., 2006). The most commonly used methods to produce PLA fibers are melt-spinning and electro-spinning (Mäkelä et al., 2002; Tsuji et al., 2006; Li and Yao, 2008). Significantly improved interfacial bonding can be achieved in materials where both matrix and reinforcing elements have the same chemical structure (Törmälä, 1992). For example, SRCs consisting of oriented PLA fibers surrounded by a PLA matrix have improved strength and rigidity compared to non-reinforced PLA (Tormala et al., 1988; Majola et al., 1992; Wright-Charlesworth et al., 2005).

To control the impact performances, molecular orientation of amorphous poly(D,L-lactide) (PDLLA) chains was carried out through injection moulding techniques at *T* < *T_g_* or by non-conventional shear controlled orientation by injection moulding (SCORIM) process in which the melt is cooled under oscillating shear conditions. The latter allowed getting oriented PLA-based materials, leading to the elaboration of degradable devices with much improved mechanical properties compared to non-oriented materials (Grijpma et al., 2002). The brittle fracture mechanism of PDLLA via crazing changed from a fragile to a ductile energy dissipation mechanism upon orientation. Consequently, a significant increase in impact strength was obtained. In comparison to the brittle tensile behavior of un-oriented PDLLA, a much more ductile behavior was observed. This increase in toughness was not accompanied by a decrease in tensile strength and stiffness, as it is generally in the case of plasticization and rubber modification. Due to orientation of the polymer chains in the direction of testing, fibrillation took place during the fracture process. Growing cracks got stopped in the anisotropic structure, and catastrophic failure could be postponed. The mechanical data are summarized in Table 2. However, in the perpendicular direction to the orientation, mechanical properties are much poorer and must be taken into account. The effects of operative SCORIM parameters were also investigated. The correlations between processing, morphology and mechanical properties of SCORIM-moulded PLLA were established and compared with conventional injection moulded CIM PLLA (Ghosh et al., 2008). The level of molecular orientation was assessed indirectly by hot recoverable strain HR test. The fracture surface-morphology assessed by optical microscopy and SEM technique showed that, at low mould temperature, the level of molecular orientation increased with shearing time. The SCORIM processing changed the typical heterogeneous skin–core morphology of CIM into a near homogeneous oriented structure. The extent of core-fibrillation increased with shearing time. Under the three-point flexural test, the higher oriented PLLA exhibited dual fractures where the crack initiation started in the skin and transferred to oriented core fraction without decreasing the modulus. At high mould temperatures, the orientation increased steadily with shearing time. However, the level of molecular orientation was lower than the corresponding low mould temperature conditions. The orientation of core-fraction increased steadily with shearing time. Depending on the level of molecular orientation, the SCORIM-processed PLLA products showed four distinct types of fracture surfaces under three-point flexural test: (i) the un-oriented core failed through crazing; (ii) the sub-skins failed either in smooth, rough or fibrillated fracture surfaces depending on the level of molecular orientation; (iii) the less oriented core failed with fibrillation through pronounced plastic deformation; and (iv) the highly oriented skins failed with smooth surface. All the SCORIM-processed PLLA exhibited higher toughness and higher maximum stress compared with conventional injection-moulded PLLA (Table 2). The overall increments in maximum stress and toughness were of 134% and 641%, respectively. The increase in maximum stress and toughness were higher in low mould temperatures (30°C) in contrast to high mould temperature temperatures (50°C). Unlike the traditional blending technique, the increments in mechanical performances were achieved without sacrificing the stiffness. The mechanical behavior namely toughness and maximum stress of PLLA processed by SCORIM could be tailored by controlling the melt stage, the in-mould shearing time and the cooling conditions. In another study (Bigg, 2005), biaxial orientation of PLLAs chains by extrusion induced a 5–10-fold increase in elongation and enhanced tensile strength at break, tensile toughness and tensile modulus (Table 2). The mechanical processing of PLA (injection and extrusion/injection) as well as annealing of processed materials were studied in order to analyse the variation of its chemical structure, thermal degradation and mechanical properties (Carrasco et al., 2010). Processing of PLA yielded a decrease of its molecular weight and melt-viscosity due to chain hydrolysis. PLA crystal structure was significantly recovered after annealing. The authors also confirmed by proton NMR techniques that the chemical composition of PLA did change after processing, and the proportion of methyl groups from PLA matrix increased, more likely indicating the presence of a different molecular environment. The mechanical behavior was altered as well (Table 2). After annealing, the samples showed an increase in Young's modulus (5–11%) and in yield strength (15–18%), which had been explained by the higher degree of crystallinity of annealed materials, with its subsequent decrease in chains mobility. Extruded/injected materials showed a significant increase in elongation at break (32–35% higher), compared to injected materials. It is ascribed to the presence of low molecular-weight chains at high contents, due to hydrolysis reactions in reprocessed materials.

In general, the modification of chain orientation and crystallinity for PLA-based materials can improve its ductility and impact resistance to some extent. Some processing techniques may contribute efficiently to toughening PLA, without compromising its tensile properties. Orientation of chains by injection moulding and especially injection moulding with macroscopic oscillating shear force resulted, for instance, in an enhancement of tensile, Izod and Charpy impact in the orientation direction. In order to increase the crystallinity of PLA blends and therefore tune its mechanical properties, some routes may be considered (Battegazzore et al., 2011):

– *By chain orientation under stress;*
– *By applying thermal treatments (quenching and/or annealing);*
– *By minimizing the amount of the other lactide and mesolactide in the lactide used as the major monomer*. The crystallinity and crystallization rate of PLA decrease as the purity decreases. The crystallization half-time was found to increase by roughly 40% for every 1 wt.% increase in the mesolactide content of the polymerization mixture (Kolstad, 1996). In addition, it is known that a co-monomer content higher of 7 wt% with polymeric chains leads to an amorphous polylactide.;
– *By playing with the moulding conditions, in particular moulding temperature and cooling time*. Even at high L-lactide content, PLA crystallization is typically too slow to develop significant crystallinity unless it is induced by strain like processes used to manufacture bottles. In processes such as injection moulding, where the orientation is limited and the cooling rate is high, it is much more difficult to develop significant crystallinity and therefore formulation or process changes are required.;
– *By adding nucleating agent*.

Nevertheless, these techniques are not very industrially considered because they require increasing the processing time. In addition, studied alone, their influences are usually marginal and the resulting increase of toughness properties is insufficient [but sometimes quite enough because excellent stiffness-toughness balance was achieved in some cases (Gamez-Perez, 2010)] to satisfy the requirement of most applications. However, the combination of these factors with others such as compounding strategies (that will be discussed further) may bring more added-values in terms of the enhancement of PLA's mechanical properties and constitute more prospective routes to improve them.

## Approaches to incorporating soft components into PLA matrix via compounding/blending

Blending polymers is as old as the polymer industry itself. Interestingly, using blending approach, PLA can be readily impact-modified, plasticized, filled, chemically modified and reactive blended and processed like many of other conventional polymers. There are two main ways to improving the ductility and the toughness of PLA materials namely through plasticization or incorporation of soft/rubbery polymers. Plasticization makes possible to achieve improved processing behaviors for polymeric materials, while providing better flexibility in the end-use product. As far as blending is concerned, blending PLA with immiscible polymers produces a new type of polymeric materials with different properties, in which each polymeric partner provide its own feature. Because of their impact-absorbing ability when well-dispersed with the convenient particle size distribution, rubbers should act as stress concentrators at many sites throughout the material. Therefore, they impart great ductility and impact strength to the material, resulting from dissipative micromechanisms initiated by the rubber particles. All of these phenomena are dependent on the deformation, toughening and fracture mechanisms, namely crazing, shear yielding, cavitation, or debonding as mostly reported in the literature (Kambour, 1973; Michler, 1989; Wu, 1990; Könczöl et al., 1992; Ikeda, 1993; Dompas et al., 1994; Lu et al., 1997; O'Connell and McKenna, 2002; Narisawa and Yee, 2006; Bucknall, 2007; Seelig and Van Der Giessen, 2009):

1. *Crazing mechanism* can be initiated in a material when the stress or hydrostatic tension is locally concentrated at a defect which can be a notch, voids, in-homogeneities or rubber particles. Therefore, interpenetrating micro-voids and highly drawn elongated micro-fibrils called *tufts* (usually a fraction of 1 μm in length, depending on the molecular weight of a polymer, several nanometers in diameter, and confined to a small volume of the material), are formed giving rise to macroscopic highly localized zones of plastic dilatational deformation (Kramer and Berger, 1990). Under sufficient mechanical loading, the local stress exceeds a critical value. Thus, the micro-fibrils elongate until breaking and cause the micro-voids growth and coalescence turning into micro-cracks. Crazing mechanism is dilatational in nature and consumes the predominant part of fracture energy in the case of many thermoplastics. Accordingly, crazing is to some extent a precursor to macroscopically brittle failure and is view as a damaging mechanism in the case of brittle polymers when the craze evolution into a micro-crack cannot be arrested. However, when blended with the brittle matrix, the rubbery impact modifier particles have two separate effects but equally important features as a response to load application. They first concentrate locally the stress where craze initiation takes place. The crazes then grow perpendicularly to the maximum applied stress direction. In a second step, the surrounding rubber particles play the role of “craze terminators,” preventing the generation of micro-cracks. The result is that a large number of small crazes are formed, in contrast with the small number of large crazes (micro-cracks) within the same polymer matrix in the absence of rubber particles. This multiple crazing that occurs throughout a comparatively large volume of rubbery-modified material explains the high energy absorption in fracture tests and the extensive stress whitening that accompanies deformation and failure (Perkins, 1999). Some matrices tend to craze because of low entanglement density while high molecular weight is needed to stabilize crazes.
– *Shear yielding mechanism* is highly localized plastic deformation characterized by appearance of oriented shear bands under uniaxial tension at 45°C to the direction of the applied stress. Shear yielding occurs approximately at constant volume while initiation of shear bands is affected by the hydrostatic tension (mean stress). In ductile polymers, shear-yielding is usually the major energy absorbing mechanism. There are also few polymers such as acrylonitrile butadiene styrene (ABS) and rubber-toughened PMMA that exhibit both shear yielding and crazing mechanisms. When the craze initiation stress of the matrix is lower than the yield stress, a polymer will tend to craze; if the yield stress is lower than the craze initiation stress, the matrix will fail by shear-yielding. Mixed crazing and shear yielding tends to occur when the craze initiation stress and the yield stress are comparable or when interactions occur between crazes and shear bands.
– *Cavitation* is void-expansion, which can occur in the matrix (generally coupling with crazing) or initiate inside the rubber particles, which is generally characterized by viewing stress-whitening zones. The essential conditions for void growth is an energy balance between the strain energy relieved by cavitation and the surface energy associated with the generation of a new surface. Cavitation is a precursor to other toughening mechanisms, thereby relieving the hydrostatic strain energy and initiating shear yielding of the matrix. It is assumed that internal rubber cavitation is an instantaneous process, which cannot occur for very small particles (less than 200 nm). In other words, rubber-cavitation mechanism is favored by increasing the particle size within rubber toughening materials or by decreasing the crosslinking density (which can suppress cavitation).
– *De-bonding* is the energy-dissipation due to the interfacial failure. The interface between the phases influences the final blends properties by efficient stress transfer between the two phases. However, interfacial de-bonding can be thought of as a secondary toughening mechanism being more important as a trigger for other induced mechanisms like shear yielding. Accordingly, low interfacial adhesion easily results in premature interfacial failure and hence rapid and catastrophic crack propagation, whereas very strong adhesion is unfavorable for de-bonding and also delays the occurrence of matrix yielding, involving the matrix-particle interface as an important factor that we need to control for optimum energy dissipation.

Toughening mechanisms and competition between both modes of fracture are mainly governed by a variety of factors such as mode of loading, environment, processing conditions, composition and behavior of the matrix, relaxation behavior of the dispersed phase, rubber content, blend morphology, rubber-matrix adhesion, etc. Being a suitable processing technique, reactive extrusion for instance, represents a unique tool to manufacture biodegradable polymers upon different types of reactive modification in a cost-effective polymer processing (Michaeli et al., 1993; Mani et al., 1999). This technique enhances the commercial viability and cost-competitiveness of polymer materials, in order to carry out not only melt blending, but also chemical reactions including polymerization, grafting, compatibilization, branching, functionalization… (Michaeli et al., 1993; Mani et al., 1999). The *in situ* chemical modification of PLA by reactive extrusion has proven to be an effective promising way to elaborate tougher PLA-based materials with improved stiffness-toughness balance compared to neat PLA as it will be detailed later. Here, the forthcoming paragraphs will report the recent investigations on simple plasticization of PLA and blending PLA with rubbery/soft materials.

### Compounding with plasticizers—miscible to partially miscible blends

Plasticization is widely used to improve the polymers processability and/or other properties according to specific applications. Plasticizers can act by altering the intermolecular interactions among the host polymer chains to other interactions between the macromolecules and the plasticizer. This promotes conformational changes, resulting in increased mobility of plasticized chains. The Standard ISO427 (1988) define a plasticizer as being as a low or negligible volatility component, which is once incorporated to a plastic material, lowers its softening interval temperature, facilitates its processability and increases its flexibility and ductility. Its behavior can be explained by decreasing the viscosity of the molten plasticized polymer, the glass transition temperature and the elastic modulus of the plasticized materials. The evolution of the elongation at break can be also related to the ductility of a polymer and give information about the plasticization extent of polymers.

To be suitable with PLA, a plasticizer should fulfill the following characteristics (Liu and Zhang, 2011):

– To have an optimum molecular weight and loading level to be miscible with the polymer matrix. Miscibility of plasticizers in a polymer matrix is evaluated by solubility parameters (δ) and magnitude of interaction parameters (χ_*T*_) (Pillin et al., 2006);
– Significantly lower the *T_g_* of PLA and thus enhance tensile toughness;
– Preferably bio-sourced and biodegradable;
– Non-volatile;
– Non-toxic;
– Exhibit minimal even more negligible leaching/migration phase separation from the polymer matrix during ageing.

Many classes of plasticizers were reported by Liu and Zhang and will be discussed in the forthcoming part as follows (Liu and Zhang, 2011):

– Monomeric or small molecule plasticizers;
– Oligomeric and polymeric plasticizers;
– Mixed plasticizers.

In the review, a special emphasis is made on the impact behavior of plasticized PLA, because it has not received enough attention in the literature.

#### Monomeric or small molecule plasticizers

Many small molecules/monomeric plasticizers have been studied in order to evaluate their plasticization efficiency and their influence on the overall physical properties of PLA (Table 3). The optimal plasticizer content has to take into account the molecular weight, solubility δ and interaction parameters χ_*T*_. For instance, most of the studies showed that between 10 and 20 wt.% of plasticizer content in PLA, all studied citrate esters (TEC, TBC, ATEC, ATBC) results in a higher elongation and lower *T_g_* for the as-plasticized PLA materials compared to neat PLA.

**Table 3:**
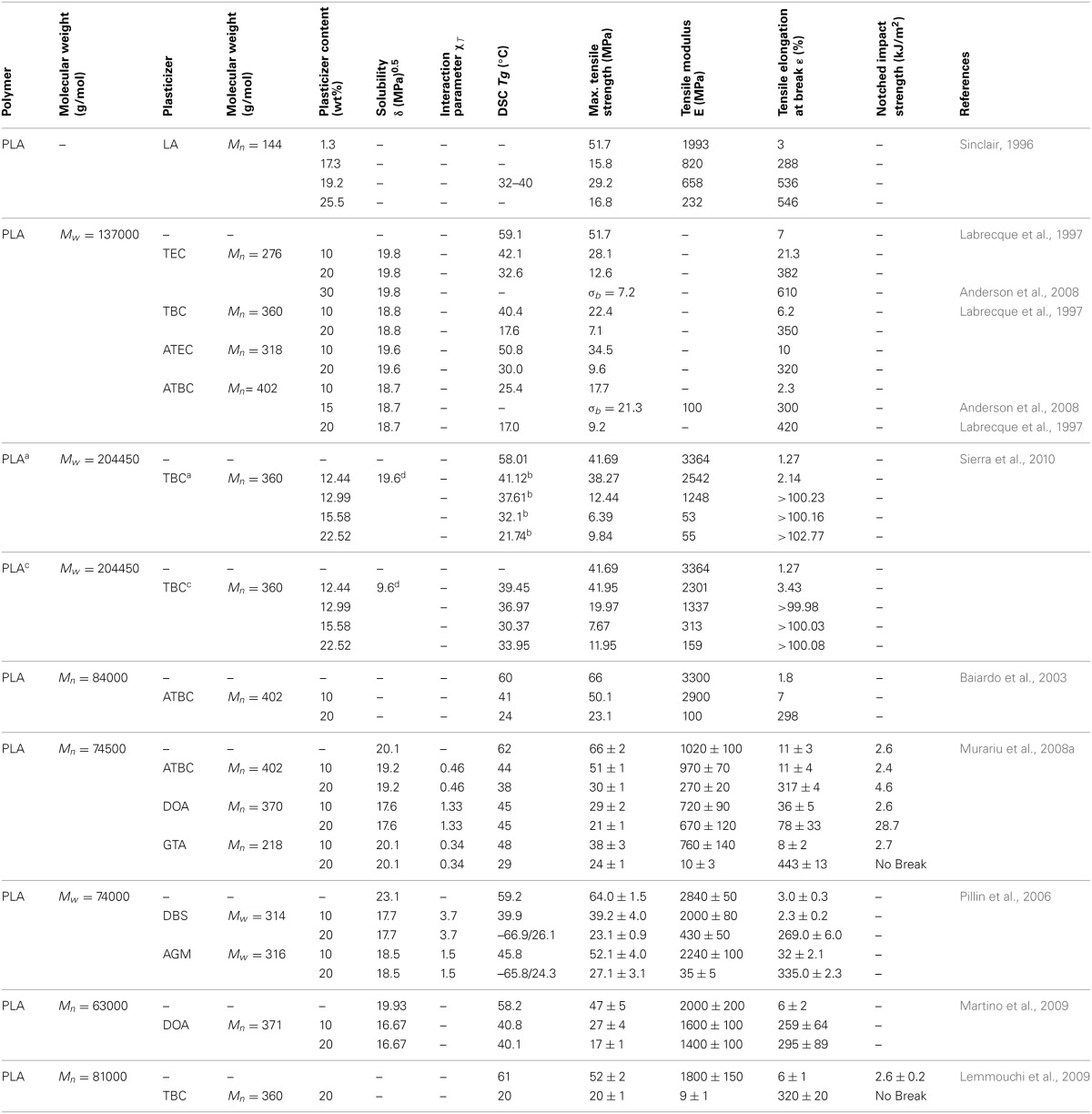
**Plasticization effects on PLA with some monomeric plasticizers [Copyright ©(2011) Wiley and Sons; used with permission from Liu and Zhang (2011)]**.

*^a^Material aged for 10 days*.

*^b^Material aged for 20 days*.

*^c^Material aged for 24 days*.

*^d^(Ljungberg and Wesslén, 2003)*.

*LA, Lactide; TEC, Triethyl citrate; TBC, Tributyl citrate; ATEC, Acetyl triethyl citrate; ATBC, Acethyl tributyl citrate; DOA, Dioctyl adipate (Bis(2-ethyldhexyl) adipate); GTA, Glycerin triacetate (Triacetin); DBS, Dibutyl sebacate; AGM, Acetyl glycerol mono–laurate; σ_b_, Tensile strength at break*.

Among the monomeric or small molecule plasticizers studied in the literature, lactide monomer (LA) possesses the best plasticization efficiency for PLA. However, due to its low molecular weight compared to the others, lactide tends to migrate toward the PLA surface. Therefore, the toughness of plasticized PLA tends to be reduced with time (Jacobsen and Fritz, 1999). LA can also volatilize during melt processing because of its low boiling point (~120°C). In terms of good stiffness-toughness balance, Dioctyl adipate (DOA) seems to be the most efficient one by significantly enhancing elongation with a slight depression of tensile modulus (Martino et al., 2009). The plasticizing efficiency of ATBC was higher compared to the others citrate-based plasticizers. Generally, the miscibility of plasticizers with a polymer decreases with increasing molecular weight of the plasticizers. Small molecule plasticizers are usually more efficient than larger ones in order to lower the host polymer's *T_g_* because the mixing entropy is higher in the case of low *M_w_* plasticizers. However, because of their low boiling point, small molecule plasticizers usually evaporate during melt processing (Labrecque et al., 1997; Ljungberg and Wesslén, 2003; Ljungberg et al., 2003; Martino et al., 2009) and have also a strong tendency to migrate toward the surface of the polymeric material (Ljungberg and Wesslén, 2003; Ljungberg et al., 2003, 2005; Martino et al., 2006). The driving force of the migration is ascribed to the enhanced crystallization ability of plasticized samples. Consequently, the ability of PLA to accommodate the plasticizer in the amorphous PLA phase diminishes (Ljungberg and Wesslén, 2002; Ljungberg et al., 2003, 2005; Martino et al., 2006; Pillin et al., 2006). In addition to the loss of the material toughness (plasticized PLA regains part of the brittleness of neat PLA); the plasticizer migration can, for example, contaminate the food or beverage in contact with plasticized PLA in food packaging applications. All monomeric plasticizers should be added in the range of 5–25% (depending on the plasticizer itself) in order to reduce the migration to the maximum, to maintain the optimum balance between tensile modulus, strength and elongation at break and reduce significantly the glass transition temperature of the host polymer. However, monomeric plasticizers cannot fulfill these requirements due to their high tendency to migrate and evaporate. In this regard, researches had been more widely focused on oligomeric and polymeric plasticizers.

#### Oligomeric and polymeric plasticizers

The common way to reduce plasticizers' migration and evaporation is to increase their molecular weight in such a way to retain their miscibility with the polymer matrix at the same time. In this respect, many researchers have investigated the effect of some oligomeric and polymeric molecules as plasticizers for PLA (Table 4).

**Table 4:**
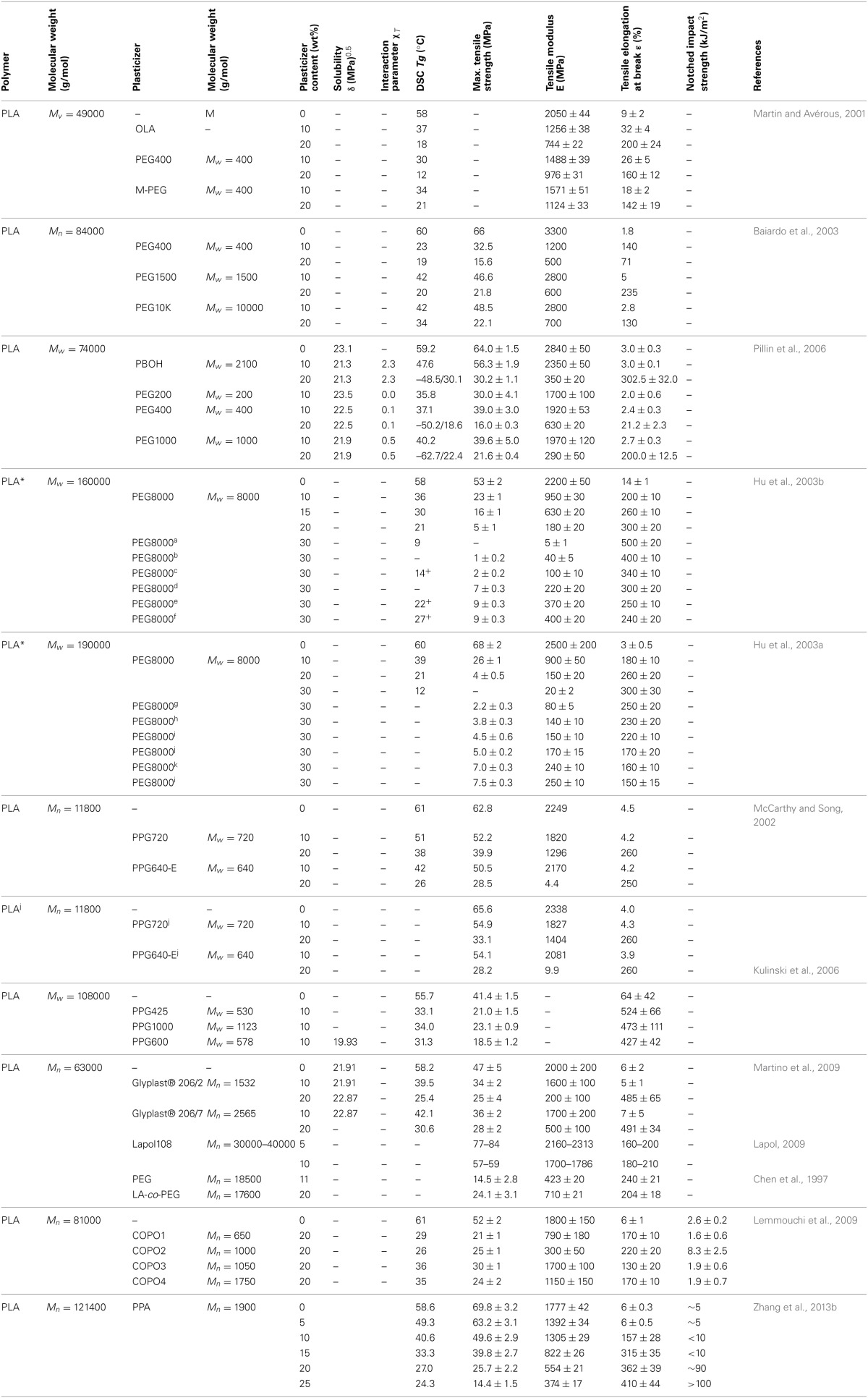
**Table molecular weight and solubility parameters (δ) of some oligomeric and polymeric plasticizers and their Plasticization effects on PLA [Copyright © (2011) Wiley and Sons; used with permission from Liu and Zhang (2011)]**.

*An update of the Table 4 has been made by Kfoury et al. in the frame of this review*.

^*^with low stereoregularity, ^+^T_g_ measured from E” in DMA

*^a^aging for 6 h*.

*^b^aging for 30 h*.

*^c^aging for 120 h*.

*^d^aging for 500 h*.

*^e^aging for 1800 h*.

*^f^aging for 2 h*.

*^g^aging for 10 h*.

*^h^aging for 24 h*.

*^i^aging for 120 h*.

*^j^aging for 720 h*.

*^k^aging for 3000 h*.

*LA, lactide; OLA, oligomeric lactic acid; PEG, poly(ethylene glycol), the subsequent number represents its nominal molecular weight; M-PEG, poly(ethylene glycol) monolaurate; PBOH, poly(1,3-butanediol); PPG, poly(propylene glycol), the subsequent number represents its nominal molecular weight; PPG-E, epoxy capped poly(propylene glycol); GlyplastVR 206/2 and GlyplastVR 206/7, two kinds of commercial polymeric adipates with molecular weight of 1532 and 2565 g/mol, respectively; COPO1 and COPO2, two kinds of AB-type block copolymers of DLLA and either PEG350 monomethyl ether or PEG750 monomethyl ether, that is, PDLLA-b-PEG350 (10/4, molar ratio of D,L-LA monomer to PEG350 used in the feed) and PDLLA-b-PEG750 (10/4, molar ratio of D,L-LA monomer to PEG750 used in the feed); COPO3, ABA-type block copolymer of PDLLA and PEG400, that is, PDLLA-b- PEG400-b-PDLLA (10/2, molar ratio of D,L-LA monomer to PEG400 used in the feed); COPO4, 3-star-(PEG-b-PDLLA) block copolymer (10/1.3, molar ratio of D,L-LA monomer to 3-star-PEG used in the feed); COPO5, 4-star-(PEG-b-PDLLA) block copolymer (10/1, molar ratio of D,L-LA monomer to 4-star-PEG used in the feed); PPA, Poly(1,2-propylene glycol adipate)*.

**Figure 2:**
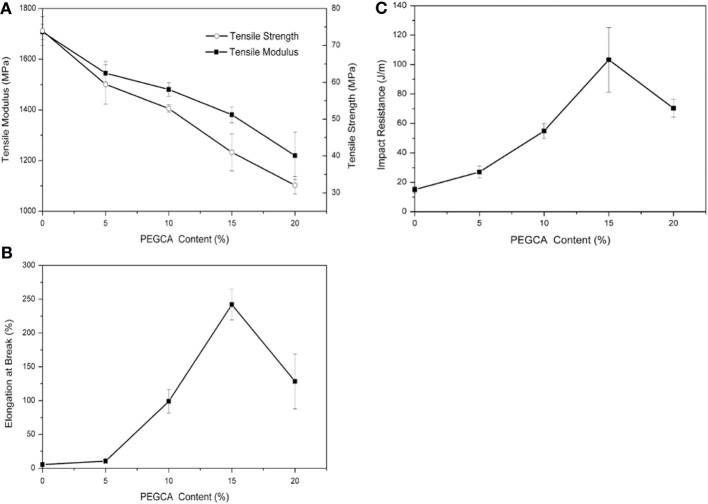
**(A)** Tensile modulus, tensile strength, **(B)** elongation at break, and **(C)** of neat PLA and PLA/PEGCA blends (Gui et al., 2013, original copyright with kind permission from Springer Science and Business Media).

For 20 wt% plasticizer content, ABA-type block copolymer of PDLLA and PEG400, that is, PDLLA-b- PEG400-b-PDLLA (10/2, molar ratio of D,L-LA monomer to PEG400 used in the feed) (COPO3) and poly(propylene glycol) (PPG720) provide a good stiffness-toughness balance. PPGs 425, 600 and 1000, Glyplast® 206/2 and Glyplast® 206/7 have a better plasticizing efficiency compared to the others. Adipates-based plasticizers are miscible with PLA until a critical concentration reached in function of the molar mass of adipate. A remarkable increase in elongation was achieved when the concentration of plasticizer reached 10 wt%, whereas the decreases in elastic modulus and tensile stress were noted for all the plasticizers investigated. Very recently, it has been shown that PLA can be efficiently plasticized and toughened by melt-blending with poly (1,2-propylene glycol adipate) (PPA) (Zhang et al., 2013b). Thermal and dynamic mechanical analysis revealed that PPA was partially miscible with PLA. In addition, morphological investigation of PLA/PPA blends showed that PPA was compatible with PLA. As a result, with the increase of PPA content (5–25 wt%), the blends showed a decrease in the tensile strength and the Young's modulus (Table 4); but the elongation at break and the impact strength dramatically increased due to the plastic deformation. The Izod notched impact strength reached 90 J/m when the PPA amount was of 20 wt%, and even exceeded 100 J/m when PPA amount was of 25 wt%. The plasticization effect of PPA was also highlighted by the lowering of dynamic storage modulus and viscosity in the melt stage of the blends compared with neat PLA. In another recent study, Gui et al. have successfully toughened PLA by melt-blending with poly(ethylene glycol-co-citric acid) (PEGCA) (Gui et al., 2013). The addition of PEGCA to PLA lowered the viscosity and the glass transition temperature of the resulting material. PEGCA was partially miscible in PLA and the blends exhibited a phase-separated morphology. The ductility and toughness of PLA were significantly improved in the presence of PEGCA. Whereas the impact resistance (Figure 2C) and the elongation at break (Figure 2B) of the blends were remarkably higher than those of neat PLA, the tensile and flexural strength and modulus of the blends (Figure 2A) monotonically dropped with increasing PEGCA content.

Hassouna et al. investigated new plasticization ways based on low molecular bio-plasticizers to improve the ductility of PLA. Grafting reactions between anhydride-grafted-PLA (MA-*g*-PLA) copolymer with hydroxyl-functionalized citrate plasticizer, i.e., tributyl citrate (TbC) (Hassouna et al., 2012), and poly(ethylene glycol) (Hassouna et al., 2011) were carried out through reactive extrusion. All plasticizers drastically decreased the *T_g_* of PLA due to the mobility gained by the polymer chains within the plasticized blends. Regardless the nature of the plasticizer, the elastic modulus and yield stress decrease, while the ultimate strain increases for plasticized PLA. Very recently, we have investigated a novel and efficient pathway to chemically modify PLA in the presence of “reactive” polyethylene glycol (PEG) derivatives via reactive extrusion (Kfoury et al. *Tunable and durable toughening of polylactide materials via reactive extrusion. Submitted*). In this purpose, polyethylene glycol methyl ether methacrylate (MAPEG) and polyethylene glycol methyl ether acrylate (AcrylPEG) were melt-mixed and extruded with PLA in the absence and in the presence of a free-radical di-tertiary alkyl peroxide, 2,5-dimethyl-2,5-di-(tert-butylperoxy)hexane (Luperox101 or L101). Molecular characterization revealed that in the case of PLA/MAPEG/L101 blends (79.5/20/0.5 wt/wt/wt), about 20% of the initially introduced MAPEG can be grafted onto PLA chains. The remaining fraction (80%) of the plasticizer was a mixture of unreacted/monomeric and “homo-oligomerized” MAPEG. As a result, an efficient plasticization effect was evidenced by a significant lowering of the glass transition temperature (*T_g_*) and storage modulus E' as well as by a drastic increase of the tensile elongation at break of approximately 70 times as compared to neat PLA. More interestingly, in the case of PLA/AcrylPEG/L101 (79.5/20/0.5 wt/wt/wt), up to 65% of the initially introduced AcrylPEG reacted and was grafted onto the PLA chains. The remaining non-grafted AcrylPEG completely homo-oligomerized. As a result, an efficient toughening effect of the resulting materials was reached. This was especially marked by a drastic enhancement of the impact strength, ~36 times, and a significant improvement of the elongation at break, ~63 times.

Lapol®108 is a renewable bioplasticizer of PLA that can be processed using standard processes such as injection moulding, extrusion coating, thermoforming, and cast films (http://www.lapol.net/). It promotes toughness and flexibility without sacrificing modulus, while minimizing the reduction of glass transition temperature. For the lowest plasticizer content (5–10 wt%), the bioplasticizer Lapol®108 seems to be the most convenient one to maintain a good stiffness-toughness balance among this list of investigated plasticizers (Table 4). Interestingly, the new Lapol® HDT additive used for increasing the heat deflection temperature of PLA is now available at pilot-production. For many high-performance applications, using PLA requires a high temperature resistance to deformation and deflection, i.e., a heat deflection temperature higher than 100°C. Compounding 20 wt.% of Lapol® HDT with PLA 3001D, 4032D, or 7000D can increase the heat deflection temperature of unannealed PLA from 55°C to about 160°C. This capability greatly expands the potential uses and applications to PLA. This increased heat-performance is achieved without adding inorganic fillers or other additives, although these additions may further enhance some other properties. Table 5 shows typical flexural properties data for a blend of 20% Lapol® HDT in PLA compared to commercially available neat and annealed PLA.

**Table 5:** **Comparison of flexural properties of Lapol® HDT blends vs. PLA (unannealed and annealed) (From http://www.lapol.net/)**.

**Flexural properties**	**Modulus (MPA)**	**HDT^b^ (°C)**
PLA	3300	55
Annealed PLA^a^	3800	155
20% Lapol HDT in PLA	3800	165

a *PLA was annealed for 10 min at 110°C*.

b *Heat deflection temperature is measured using a thermomechanical analyzer using a load of 0.2–0.3 N*.

PLA-based blends containing Lapol® HDT exhibit similar or higher flexural modulus than commercially available PLA (annealed and unannealed). Lapol® HDT may be compounded with an impact modifier to tailor the properties of PLA for specific applications.

Globally, these studies show that oligomeric and polymeric plasticizers are in general less efficient than monomeric ones in order to improve the elongation and reduce the glass transition temperature of resulting blend. However, they have more tendencies to give better stiffness-toughness balance for PLA material compared to small molecule plasticizers. Based on their complementary advantages, the combination of small molecule plasticizers with polymeric or oligomeric ones was also attempted in the literature.

#### Mixed plasticizers

These mixed plasticizers combine an oligomeric or polymeric plasticizer with a small molecule plasticizer. Therefore, they should lead to a medium level of depression in *T_g_* and more balanced mechanical properties (elongation, modulus and strength) than the individual plasticizers. Some plasticizer combinations were studied. They are reported in Table 6.

**Table 6:**
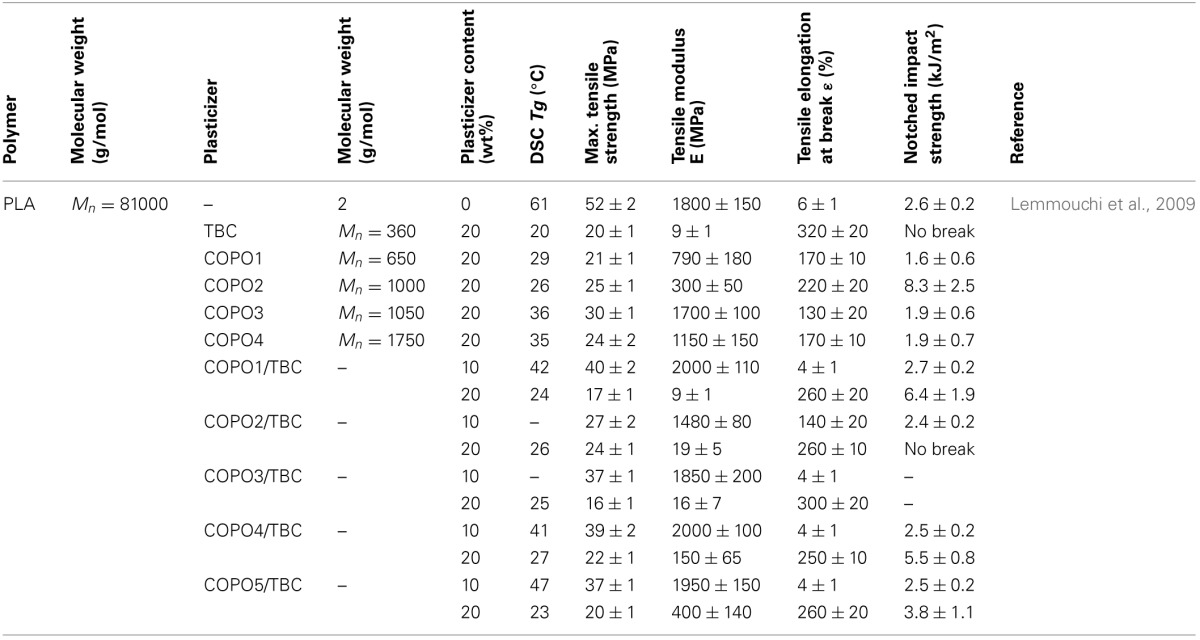
**Summary of effects of some mixed plasticizers on mechanical properties of PLA [Copyright © (2011) Wiley and Sons; used with permission from Liu and Zhang (2011)]**.

*TBC, tributyl citrate; COPO1 and COPO2, two kinds of AB-type diblock copolymers of PDLLA and either PEG350 monomethyl ether or PEG750 monomethyl ether, that is, PDLLA-b-PEG350 (10/4, molar ratio of D,L-LA monomer to PEG used in the feed) and PDLLA-b-PEG750 (10/4, D,L-LA monomer to PEG molar ratio used in the feed); COPO3, ABA-type triblock copolymer of DLLA and PEG400, that is, PDLLA-b-PEG400-b-PDLLA (10/2, molar ratio of D,L-LA monomer to PEG used in the feed); COPO4, 3-star-(PEG-b-PDLLA) block copolymer (10/1.3, molar ratio of D,L-LA monomer to PEG used in the feed); COPO5, 4-star-(PEG-b-PDLLA) block copolymer (10/1, molar ratio of D,L-LA monomer to PEG used in the feed)*.

In general, one can conclude on the behaviors of the plasticizers in PLA and their effect on the properties of the polymer as follows:

– The addition of 10–20% of plasticizers may be a successful way to remarkably reduce *T_g_* and improve PLA flexibility/ductility/tensile elongation at the same time.
– Substantial reductions in tensile strength and modulus are unfortunately unavoidable.
– An excessive incorporation of plasticizer leads to the saturation of the plasticizer in the amorphous phase of PLA, resulting in a migration or phase separation depending on the plasticizer nature.
– Small molecule or monomeric plasticizers are more efficient in order to improve PLA flexibility/ductility/tensile elongation and decrease its *T_g_*, but less efficient on tensile strength and modulus than oligomeric and polymeric plasticizers.
– The higher the molecular weight of the plasticizer, the lower the critical saturation concentration, at which phase separation begin to occur.
– Lower molecular weight PEGs exhibit good miscibility with PLA and result in more efficient reduction of *T_g_*. This can lead to drastic improvement in ductility and/or impact resistance of PLA at low concentrations.
– After ageing for 1 month, the mechanical properties of the plasticized PLA did not change remarkably. This result indicated that PPG and PPG-E could prevent the physical ageing and the embrittlement of PLA.
– Whilst increasing the molecular weight of the plasticizer can slow down migration rate and thus improve morphological stability of PLA materials during storage, it also decreases its solubility and plasticizing efficiency. Additionally, high-molecular weight plasticizers are keen to phase-separation because of low saturation concentrations of plasticizers.

### Compounding with flexible/soft polymers—partially miscible to immiscible blends—ways of compatibilization

The term “blending” refers to the simple mixing of polymeric materials in the molten state. During the last three decades, polymer blends have become a very important part of the commercialization of polymers because one can tailor blend compositions to meet specific end-use requirements (Baker et al., 2001). Melt-blending polymers is a much more economical and convenient methodology at the industrial scale rather than synthesizing new polymers to achieve the properties unattainable with existing polymers. However, most polymer pairs are immiscible, which can lead to phase-separated materials. The latter has often three inherent problems if the morphology and the interfaces of the blend are not well-controlled: (1) poor dispersion of one polymer phase in the other one; (2) weak interfacial adhesion between the two phases; and (3) instability of immiscible polymer blends (Baker et al., 2001). However, immiscible polymer blends are much more interesting for commercial development because immiscibility allows maintaining the good features of each polymeric component of the blend. One of the most important challenges is thereby to develop compatibilization techniques that allow controlling both the morphology and the interfaces of phase-separated blends. In general, compatibilization in physical blends is tuned by the physical interactions (hydrogen bonds, Van der Waals interactions etc.) between the blend components.

PLA has been blended with various polymers for different purposes, namely for improving its stiffness-toughness balance. A variety of biodegradable and non-biodegradable soft polymers have been used as toughness modifiers for PLA. Recently, it has been shown that new impact modifiers can efficiently strengthen/toughen brittle/stiff PLA, due to their core-shell polymeric structure (a block copolymer). They form a soft or elastomeric block having high compatibility and miscibility with the toughening polymer, and surrounded with a rigid block copolymer, usually having a high compatibility and/or miscibility with the matrix polymer. When the softer component forms a second phase within the stiffer continuous phase, it may act as a stress concentrator, which enables ductile yield and prevents brittle failure (Babcock et al., 2008). At the same time, the core is “locked in” by slight crosslinking and grafting with its shell to avoid phase-separation during blending. Moreover, the adhesion between the two phases, core-shell polymer and polymer matrix, depends strongly on the degree of miscibility of the shell polymer with the matrix, *that is*, whether they are completely miscible, partially miscible, or immiscible. However, a partial miscibility between core-shell modifiers and polymeric matrix is often necessary to obtain blends of desired impact properties. From the literature, multiple crazing initiated from the dispersed rubber phase is recognized to be one of the main mechanisms, which increases the toughness of glassy materials like polylactide-based materials (Ikeda, 1993; Bucknall, 2007; Mahajan and Hartmaier, 2012). Some authors have preferred to blend PLA with biodegradable flexible/soft polymers in order to preserve the overall biodegradability of resulting blends. Some of these blends are in this regard, finding short-term applications, namely packaging and mulch films for agriculture.

#### Flexible/soft (ε-caprolactone)-based copolymers

**Figure 3:**
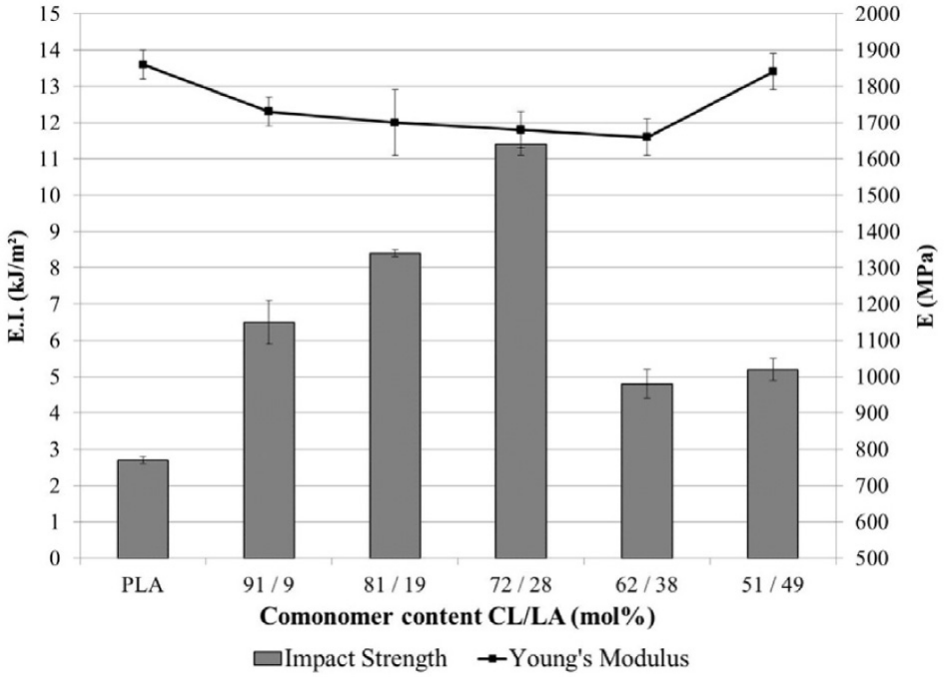
**Influence of the LA comonomer content of copolyester on the notched Izod impact strength and Young's modulus of PLA-based materials containing 10 wt.% of P[CL-*co*-LA]**. [Reprinted from Odent et al. (2013b) with permission from Elseiver].

As obtained by ring-opening polymerization of e-caprolactone, poly(e-caprolactone) (PCL) is a biodegradable and flexible/soft polyester with a melting temperature of 60°C and a glass transition temperature of −60°C. Due to the low glass transition temperature, PCL-based materials were considered as interesting impact modifiers. PCL and PLA blends have been extensively investigated over the past years. For instance, Broz et al. investigated the tensile properties of blends made of P(D,L-LA) and PCL at different content in PCL (Broz et al., 2003). Whilst the strain-at-failure decreases monotonically for PCL contents from 0.6 wt%, the modulus and tensile strength increased almost linearly with composition. This was more likely ascribed to some strengthening of the blend interface in this regime. However, DSC and NMR results suggested that PCL was able to crystallize to a certain extent within PCL/P(D,L-LA) blends, indicating that phase-separation was more pronounced under these conditions. However, as shown here, the simple melt blending of PLA and PCL usually results in a marginal toughness improvement because of their poor miscibility (López-Rodríguez et al., 2006). This can be more likely explained by the fact that PCL can readily crystallize within PLA/PCL blends, leading to the more pronounced phase-separation. Accordingly, the simple melt blending of PLA and PCL usually leads to a marginal improvement in toughness because of their immiscibility (López-Rodríguez et al., 2006; Vilay et al., 2009). In this regard, some of us have designed bio-sourced and hydrolytically degradable random copolyesters based on poly(ε-caprolactone) as a soft core component. (Co)polymerization of CL with other lactones affords an elegant way to modulate the thermal and mechanical properties of resulting PCL-based materials. The most interesting feature was that the overall crystallinity of these (co)polyesters decreased with the comonomer content, yielding rubbery-like materials at ambient temperature. When dispersed into glassy materials like PLA, it is well-known that rubbery microdomains can readily absorb the impact energy. In a first study (Odent et al., 2012), 10 wt.% of amorphous poly(ε-caprolactone-*co*-δ-valerolactone) (P[CL-*co*-VL]) random aliphatic copolyesters were thereby synthesized and investigated as biodegradable impact modifiers for commercial PLA using a microcompounder. The use of a high molar mass copolyester (*M_n_* = ca. 60,000 g/mol) with a molar composition of 45/55 mol% (CL/VL) resulted in the optimal improvement in notched Izod impact strength for compression-moulded (vs. injection-moulded) PLA materials (7 kJ/m^2^) compared to 2.5 kJ/m^2^ for PLA. According to the author, this improvement in toughness is also related to the mean size (0.7 μm) and size distribution of the dispersed copolymer micro-domains throughout the PLA matrix. In a similar study (Odent et al., 2013b), the random biocopolyester was synthetized and used as impact modifier is poly(ε-caprolactone-*co*-d,l-lactide) (P[CL-*co*-LA]). By varying the comonomer content, a phase inversion was noticed. A control of the affinity between PCL-based impact modifiers and PLA matrix gives access to a mixture of spherical microdomains with similar range of optimum particles diameter (i.e., 0.9 μm) and nanosized oblong structures, involving a combination of shear yielding and multiple crazing mechanisms. As a result, PLA blended with 10 wt.% of the CL/LA composition 72/28 mol.% displayed a maximum impact strength of about 11.4 kJ/m^2^ (Figure 3). The mean size of the rubbery micro-domains was 0.9 μm in this case.

In the case of brittle polymers, spherical microdomains act as stress reservoirs and initiate crazing upon the microdomains size, i.e., larger microdomains size than 0.5 μm are required to nucleate crazing mechanism and enhance fracture energy absorption (Donald and Kramer, 1982; Van Der Wal and Gaymans, 1999). Accordingly, an optimum particle size range (ca. 0.7–0.9 μm) for PLA toughening was identified by correlating dispersed microdomains size with notched Izod impact strength (Gramlich et al., 2010; Liu et al., 2011). Wu and al. correlated rubber particle diameter with chain structure parameter of the matrix and claimed that the optimum particle size for toughening decreased as the matrix becomes less brittle (Wu, 1990). Kowalczyk et al. reported that rubbery poly[1,4-*cis*-isoprene] microdomains within PLA-based materials initiated crazing at the early stages of deformation, immediately followed by the cavitation phenomena inside rubbery microdomains. This latter promotes further shear yielding for PLA matrix (Kowalczyk and Piorkowska, 2012). More recently, some of us have elaborated ultratough PLA-based materials by synergistically adding PLA, rubber-like poly(ε-caprolactone-*co*-D,L-lactide) copolyester and silica nanoparticles using extrusion techniques (Odent et al., 2013a). A peculiar alteration for the phase-morphology of the rubbery phase within PLA matrix was achieved by co-adding copolyester and silica nanoparticles into PLA matrix. It resulted that regularly obtained spherical nodules convert into almost continuous features after adding nanoparticles in the PLA-based melt-blend. In the latter, an enhancement of 15-fold impact strength was obtained by comparison to unfilled PLA.

The use of small molecule reactive additives during compounding has been demonstrated to be an effective way to improve the compatibility between PLA and PCL. Wang et al. (1998) investigated the tri-phenyl phosphate (TPP) as a catalyst or coupling agent for the preparation of PLA and PCL blends. The addition of 2 phr TPP to PLA/PCL (80/20, w/w) blend during processing resulted in a higher elongation (127 vs. 28%) and tensile modulus (1.0 GPa vs. 0.6 GPa) compared to the binary TPP-free blend. The balance between degradation of molecular weight and the formation of copolymer was believed to govern the final mechanical properties of the blends. Reaction time and molecular weight of PCL used were found to have remarkable effects on mechanical properties of the blends. Higher molecular weight PCL (*M_n_* = 80,000 g/mol) and medium reaction time (15 min) promoted the largest improvement of the ductility. In another study, di-cumyl peroxide (DCP) was used to promote reactive compatibilization of the PLA/PCL blends (Semba et al., 2006). The results showed again that the addition of DCP increased the ductility of the final material. Further addition of DCP beyond the optimum amount had an opposite effect on elongation. AFM observation revealed that the diameter of the dispersed PCL domains decreased with increasing DCP content. The addition of 0.3 phr DCP to the optimum ration PLA/PCL = 70/30 resulted in (1) an impact strength of 2.5 times more than that of neat PLA, (2) an improved blend compatibility, (3) an improved ultimate tensile strain (4) a yield point and ductile behavior under tensile test, and (5) mechanical properties comparable to those of HIPS and ABS. In contrast, the addition of DCP to PLA alone did not alter mechanical properties. It was suggested that DCP caused crosslinking of PLA with PCL and therefore improved interfacial adhesion. Depending on feeding procedure, addition of DCP *via* the splitting feeding method resulted in a higher reverse Izod impact strength than feeding at once through the main hopper (Semba et al., 2007). Lysine tri-isocyanate (LTI) as a reactive compatibilizer improved the compatibility of PLA and PCL, resulting in the reduction of size of PCL spherulites (Takayama and Todo, 2006; Takayama et al., 2006). Impact fracture toughness markedly improved by increasing LTI content, which was attributed to the strengthening structure of the blend as a consequence of crosslinking reactions. The compatibilizing effect of LTI was compared with four other reactive processing agents on the PLA/PCL (80/20, w/w) blends (Harada et al., 2008). The addition of 0.5 phr of each reactive agent resulted in an increase in the un-notched Charpy impact strength in the order of LTI > LDI (lysine diisocyanate) > Duranate TPA-100 [1,3.5-tris(6-isocyanatohexyl)- 1,3,5-triazinane-2,4,6-trione] > Duranate 24A-100 [1,3,5-tris(6-isocyanatohexyl)biuret] > Epiclon 725 (trimethylolpropane triglycidyl ether). It was assumed that the reaction of isocyanates group with both terminal hydroxyl and carboxylic groups of polyesters accounted for improved compatibility at the PLA/PCL interfaces and therefore the enhancement in the physical properties.

#### Polyhydroxyalkanoates (PHAs) and their copolyesters

**Scheme 1:**
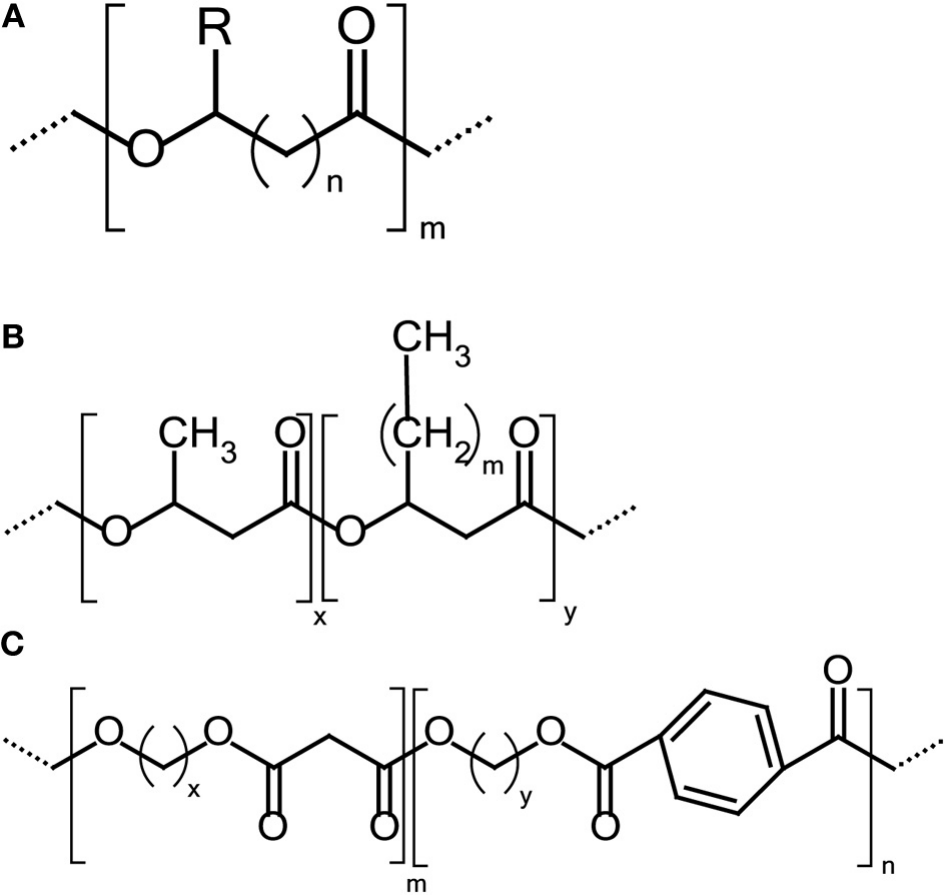
**(A)** General chemical structure of PHAs polyesters where R=hydrogen or hydrocarbon chains of up to C15 in length; *x* = 1 to 3. **(B)** The general structure of PHA copolyesters. **(C)** Chemical structure of poly(butylene adipate-co-terephthalate) (PBAT).

Polyhydroxyalkanoates (PHAs) are biodegradable polyesters produced by bacterial fermentation of sugar or lipids (Steinbüchel and Valentin, 1995; Zinn et al., 2001; Poirier, 2002; Noda et al., 2005) when nutrient shortage is present. Since the range of monomers available is impressive within this family, the mechanical properties of PHAs can range from stiff thermoplastics to elastomers dependent on their pendent alkyl chain length (Scheme 1A). However, only one grade, i.e., Nodax™, was industrially implemented by Procter and Gamble Co., which correspond to copolymers of 3-hydroxybutyrate with a small amount of 3-hydroxyalkanoate as co-monomer (Scheme 1B) (Noda et al., 2004, 2005).

In this regard, Noda et al. (2004) melt-blended PLA with a poly(3-hydroxybutyrate-co-3-hydroxyhexanoate) copolymer, i.e., NodaxH6, containing 5 mol% of 3-hydroxyhexanoate (3-HH) unit. The PLA/NodaxH6 (90/10, w/w) blend exhibited a tensile toughness of 10 times more than that of neat PLA and an elongation superior to 100%. When NodaxH6 content was less than 20 wt % in the blends, its crystallization was largely restricted and thereby NodaxH6 was dispersed as rubbery amorphous droplets within PLA, suggesting that the material was toughened by craze-initiation. Furthermore, it was interesting that the inclusion of these small amounts of PHA did not compromise the optical clarity of PLA itself.

Schreck and Hillmyer investigated the impact toughness of blends of PLLA with a NodaxH6 containing 7 mol.% of 3-HH (Schreck and Hillmyer, 2007). The PLLA/NodaxH6 (85/15, w/w) blend demonstrated a twofold increase in notched Izod impact strength (44 J/m) compared with that of PLLA (22 J/m). Ma et al. toughened PLA by melt-compounding with fully bio-based and bio-compostable poy(β-hydroxybutyrate-co-β-hydroxyvalerate) (PLA/PHBV) with high β-hydroxyvalerate content (40 mol%) (Ma et al., 2013). The blends displayed two separate glass transition temperatures and two separate phases, indicating that the PLA and PHBV were immiscible. The toughness and the ductility of PLA can be effectively improved by incorporation of 10–30 wt% of the PHBV as evidenced by a significant increase in the elongation at break and the impact toughness (Table 8). The local deformation mechanism revealed that fibrillation, partial interfacial de-bonding, cavitation and matrix yielding were involved in the toughening mechanism of the PLA/PHBV blends under impact and tensile testing conditions.

#### Biodegradable poly(butylene adipate) (PBA), poly(butylene succinate) (PBS), and poly(butylene adipate-co-butylene terephthalate) (PBAT)

Poly(butylene adipate-*co*-terephthalate) (PBAT) is a fully biodegradable aliphatic–aromatic copolyester (Scheme 1C), which is commercially available under the trade name of EcoflexVR (BASF Co.).

PBAT has similar thermal properties to those of LDPE, but exhibits higher mechanical properties, more particularly higher flexibility and ductility (elongation > 700%). Even though PLA/PBAT blend are immiscible, PBAT could be dispersed in PLA with an average particle size of about 0.3–0.4 μm without use of compatibilizers in co-rotating twin-screw extruder (Jiang et al., 2006). The mechanical properties of the different PLA/PBAT blends are reported in Table 8. It was demonstrated that the de-bonding-induced shear yield was responsible for the remarkable high extensibility of the blends. Because of weak interfacial adhesion in the blends, impact toughness was slightly improved. Interestingly, the PLA/PBAT blends are now being commercially produced by BASF Co. under the trademark EcovioVR for film and extruded foam applications.

**Figure 4:**
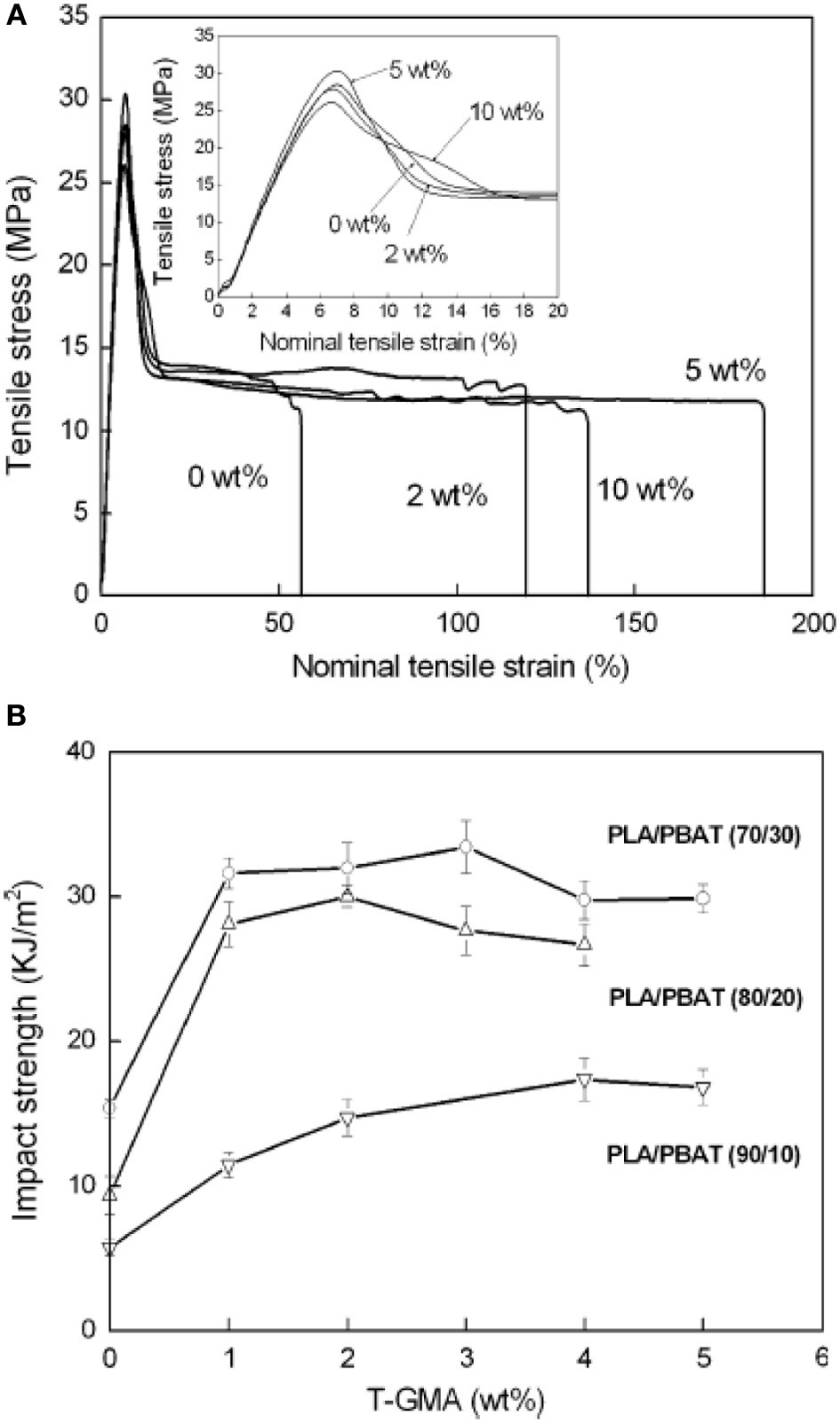
**(A)** Stress–strain curves of PLA/PBAT (90/10 wt%) blend in the presence of T-GMA. The inset gives details of stress–strain of the blends in the neighborhood of yield points. **(B)** Effect of T-GMA concentration in the PLA/PBAT blends (PLA/PBAT = 90/10, 80/20, 70/30 wt%) on impact strength. [Zhang et al. (2009a) original copyright with kind permission from Springer Science and Business Media].

**Scheme 2:**
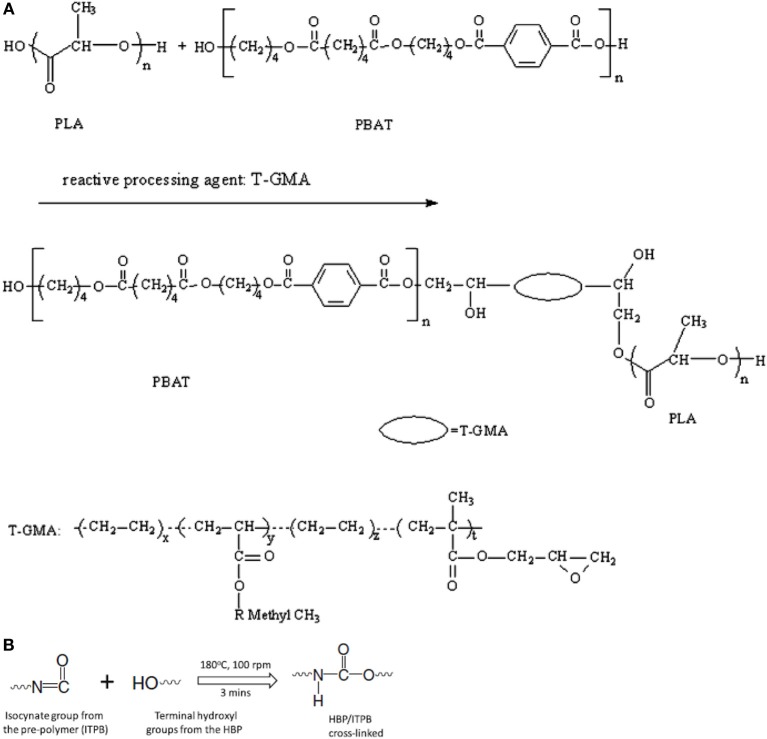
**(A)** Predicted reaction of PLA, PBAT, and T-GMA [Zhang et al. (2009a), original copyright with kind permission from Springer Science and Business Media]. **(B)** Schematic showing the proposed *in-situ* crosslinking of the terminal hydroxyl groups from HBP or PLA with isocyanate groups from the ITPB during reactive blending in extruder at 180°C [Nyambo et al. (2012), original copyright with kind permission from Springer Science and Business Media].

To improve the compatibility of PLA/PBAT blends, a random terpolymer of ethylene, acrylate ester, and glycidyl methacrylate (referred as “T-GMA”) was investigated as a reactive compatibilizer in melt compounding (Zhang et al., 2009a). Regardless the PLA/PBAT blends composition (70/30, 80/20 or 90/10 wt/wt), the increase of T-GMA content up to 5 wt% resulted in a great improvement of tensile nominal strain at break (Figure 4A) and the notched impact strength (Figure 4B) to reach more than 150% and 30 kJ/m^2^, respectively, approximately two times that of the uncompatibilized binary blends. These results were correlated to the good miscibility and interfacial adhesion between PLA and PBAT, leading to a shear yielding mechanism when increasing the T-GMA content. The authors attributed the better interfacial adhesion to the *in situ* reactive compatibilization phenomena (Scheme 2A).

**Figure 5:**
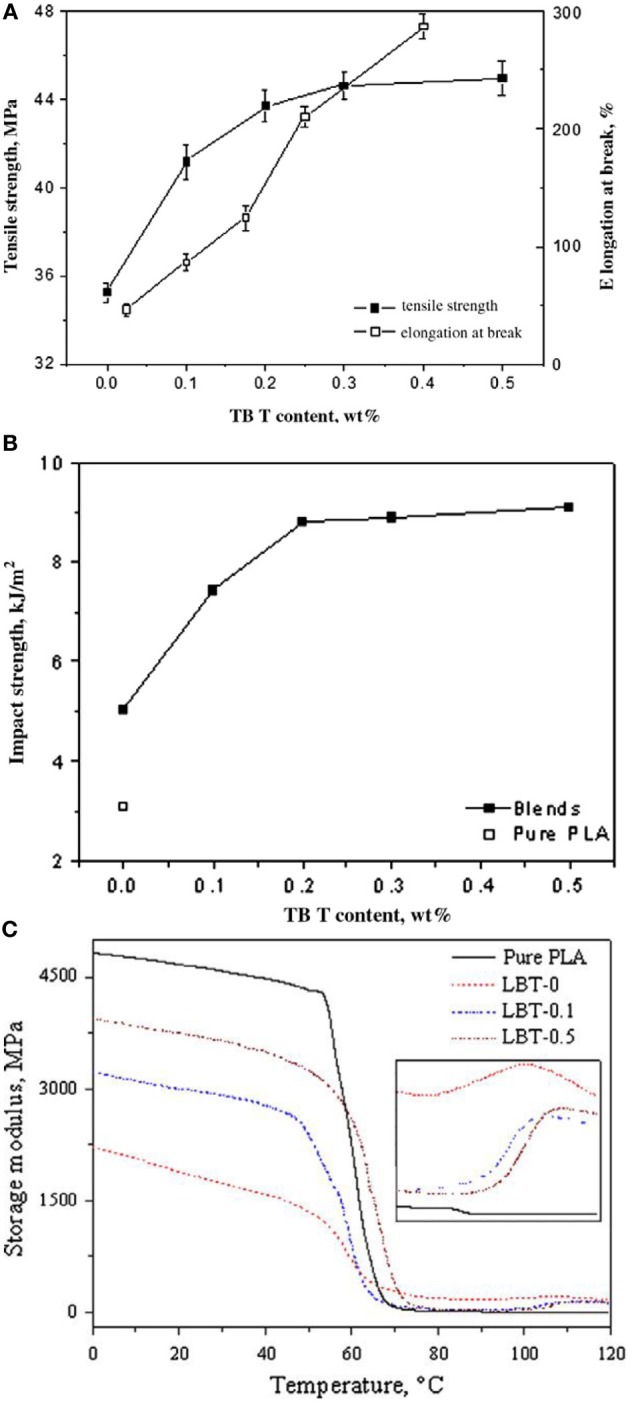
**(A)** Effect of TBT concentrations on the tensile strength and elongation at break. **(B)** Variation of impact strength of PLA/PBAT blends with TBT concentration. **(C)** Temperature dependence of storage modulus of pure PLA and its blends [Reprinted from Lin et al. (2012) with permission from Elseiver].

Lin et al. (2012) compatibilized the biodegradable blends poly(lactic acid) (PLA)/poly(butylene adipate-*co*-terephthalate) (PBAT) by *in situ* transesterification using various amounts of tetrabutyl titanate (TBT) as catalyst. The incorporation of 0.5% of TBT into PLA/PBAT blends not only improved their overall mechanical properties as well as gave values of tensile strength, elongation at break and impact strength of 45 MPa, 298% and 9 kJ/m^2^ (Figures 5A,B), respectively. It was also demonstrated that the storage modulus of the blends and glass transition temperature (Figure 5C) were enhanced compared to the binary blends free of TBT. The SEM micrographs demonstrated that the compatibility between PLA and PBAT was improved *via* transesterification during reactive melt-extrusion. The interfacial de-bonding and the yielding deformation were the most important mechanisms to improve toughness.

#### Poly(butylene succinate)s (PBS) and their copolyesters

PLA is immiscible with PBS. In some studies, a third *in situ* reactive component was incorporated to improve compatibility. PBS was melt blended with PLA without compatibilizer and using LDI and LTI as compatibilization agents (Harada et al., 2007). For all PLA/PBS compositions ranging from 90/10 to 80/20 wt.%, only the addition of 0.5 wt.% of LDI or 0.15 wt.% of LTI increased the elongation at break to more than 150%. Impact strength also increased to reach 50–70 kJ/m^2^. For PLA/PBS (80/20 wt/wt) with LTI, the un-notched samples did not break during the impact test. Furthermore, due to the addition of 0.15 phr LTI into the PLA/PBS (90/10, wt/wt) blend, the size of dispersed PBS particles was significantly reduced. Consequently, LTI was an effective reactive processing agent capable of increasing the toughness of the PLA/PBS blends. Similar results were observed by using LTI with PLA/PBSL (Vannaladsaysy et al., 2009) with effectively improved blend compatibility and higher energy dissipation during the initiation and propagation of crack growth. This results in the suppression of spherulite formation of PBSL and the formation of a firm structure made of entanglements between both PLLA and PBSL chains. DCP was also used for *in situ* compatibilization of the PLLA/PBS (80/20 wt/wt) blend (Wang et al., 2009). The uncompatibilized blend showed much higher elongation than PLLA (250 vs. 4%), but only slightly higher notched Izod impact strength (3.7 kJ/m^2^ vs.2.5 kJ/m^2^ for PLA). Addition of 0.1 phr DCP greatly increased the impact strength of the blend to 30 kJ/m^2^. Both strengths and moduli invariably decreased with increasing DCP content. It was found that the addition of DCP led to a reduction in the size of the PBS domains and improved interfacial adhesion between the PLLA and PBS phases. The toughening effect of the blends was considered to be related to the de-bonding initiated shear yielding. In a similar way, blending PLA with other polycondensates like biodegradable poly(butylene adipate) (PBA) and poly(butylene succinate) (PBS) was also investigated on the toughening effect. These results were compared with PBAT/PLA blends. Like PBAT, these (co)polyesters can be readily synthesized by melt-polycondensation (Zhao et al., 2010). As far as blends are concerned, a considerably high elongation at break with a moderate loss of strength was observed for all the blends, regardless the investigated copolyesters. For instance, the elongation at break and the impact strength increase with polyester content, until reaching maximum values (>600% and >35 KJ/m^2^, respectively) at a PBA/PBS/PBAT content of 15 and 20%, respectively. The addition of PBA/PBS/PBAT into PLA improves the toughness, but reduces the stiffness of the latter. Moreover, the crystallization ability of PLA blends can be increased by the addition of a small amount of PBS/PBA/PBAT.

#### Poly(ethylene oxide-b-amide-12) (PEBA) or Pebax®

Biosourced and biodegradable poly(ethylene oxide-*b*-amide-12) (PEBA) was used as a toughening agent for PLA *via* melt compounding. PLA/PEBA blends are an immiscible system with a two-phase morphology (Han et al., 2013). By increasing the PEBA content, the binary blends displayed a marked improvement in toughness. All PLA/PEBA blends showed a clear stress yielding on the stress–strain curves with necking when the PEBA contents varied from 10 to 30%. For the blend with 20% PEBA, the elongation at break markedly increased to 346%, corresponding to a 50-fold increase compared with the elongation at break of neat PLA. The impact strength of the blend was significantly enhanced at 20% (or more) of added PEBA as well. The maximum impact strength reached was of 60.5 kJ/m^2^, indicating that a significant toughening effect was achieved (Table 8). The phase morphology evolution in the PLA/PEBA blends during tensile and impact tests were investigated, and the corresponding toughening mechanism was discussed. Remarkably, a clear shear yielding bands perpendicular to the stretching direction and crack propagation along the tensile direction were observed during the tensile test. Moreover, the obvious plastic deformation in the blend was observed during the impact test. The shear yielding induced energy dissipation and therefore led to the improvement in toughness of the PLA/PEBA blends.

#### Polyurethane and polyamide elastomers

**Figure 6:**
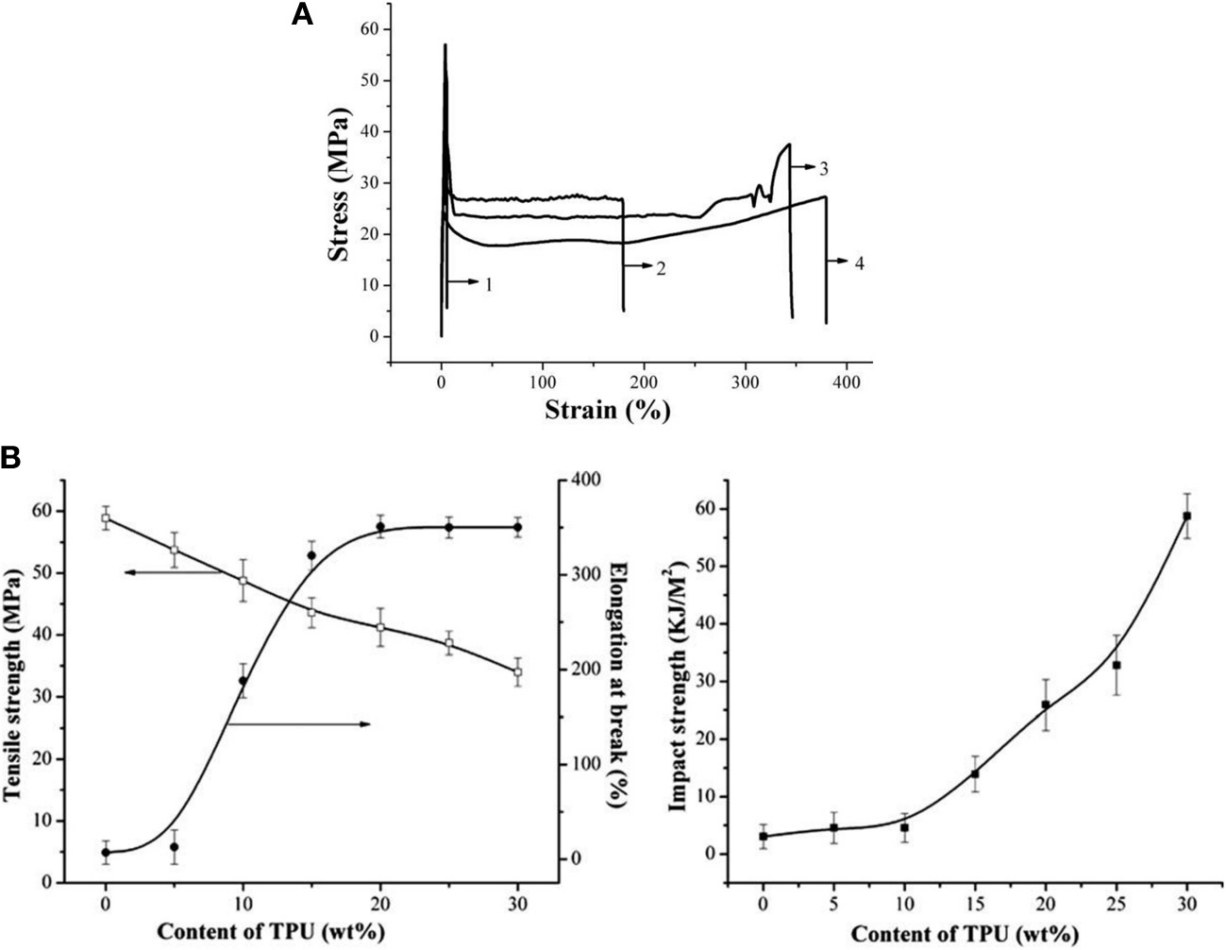
**(A)** Tensile stress–strain curves of the PLA/TPU blends: (1) PLA/TPU (100/0), (2) PLA/TPU (90/10), (3) PLA/TPU (80/20), and (4) PLA/TPU (70/30). **(B)** Mechanical properties of the PLA/TPU blends as a function of the TPU content. Copyright (2010) Wiley; used with permission from Feng and Ye (2011).

Feng et al. used a thermoplastic polyurethane (TPU) elastomer with a high strength, toughness and biocompatibility to prepare PLA/TPU blends suitable for a wide range of applications of PLA as general-purpose plastics (Feng and Ye, 2011). The morphological structure and mechanical properties of the PLA/TPU blends indicated that an obvious yield and neck formation was observed for the PLA/TPU blends (Figure 6A). The stress–strain curves of the blends exhibited an elastic deformation stress plateau, indicating the transition of PLA from brittle to ductile fracture. The elongation at break and notched impact strength for the PLA/20 wt% TPU blend reached 350% and 25 kJ/m^2^, respectively, without an obvious drop in the tensile strength (Figure 6B). The respective *T_g_*'s of PLA and TPU in the blends also shifted to intermediate values, suggesting a partially miscible system due to the hydrogen bonding formed between the chains of TPU and PLA. Spherical particles of TPU dispersed homogeneously in the PLA matrix, and the fracture surface presented much roughness. With increasing TPU content, the blends exhibited increasing tough failure thanks to the improved crack initiation resistance and crack propagation resistance. It was evident that the use of TPU greatly improved the toughness of PLA.

**Figure 7:**
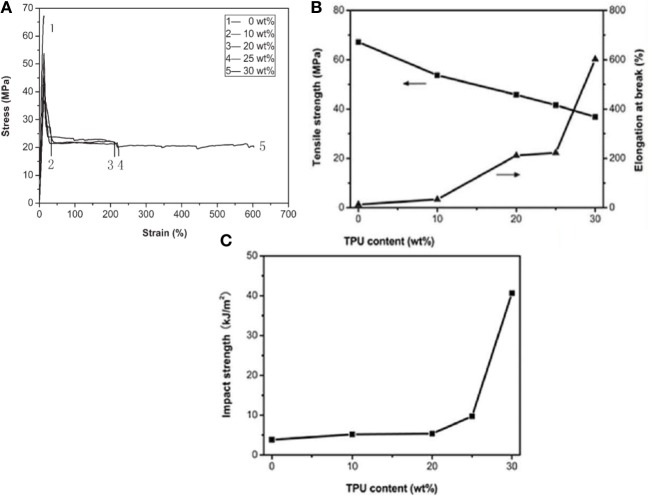
**(A)** Stress–strain curves, **(B)** Tensile properties, and **(C)** Impact strength of PLA/TPU blends with different TPU contents. Copyright (2011) Wiley; used with permission from Han and Huang (2011).

In a similar study, Han et al. also investigated the toughness effect of TPU on PLA (Han and Huang, 2011). The study of the blends morphology as a function of TPU contents showed that PLA was incompatible with TPU. The spherical particles dispersed in PLA matrix, and the uniformity decreased with increasing TPU content. There existed long threads among some TPU droplets in blend with 30 wt.% TPU. After addition of 30 wt.% TPU, the elongation at break of the blend reached about 600% (Figure 7), and the samples could not be broken up in the notched Izod impact tests at room temperature. The toughening mechanism was analyzed through three aspects, including the stress whitening, matrix ligament thickness, and observation of the fracture surface of the impacted sample. The matrix ligament thickness of the PLA/TPU blends was below the critical value, and the blends deformed to a large extent because of shear yield initiated by stress-concentrations and interfacial de-bonding. This resulted in the formation of fibers in both tensile and impact samples and the dissipation of a large amount of energy.

Some other blends with polyurethane (PU) and polyamide elastomers (PAE) were elaborated in order to study their mechanical properties (Table 7). PLA/PU blends were found to be partially miscible, and PU was dispersed in PLA within domain sizes at the submicrometer scale. It was demonstrated that matrix shear yielding initiated by de-bonding at the matrix/particle interface was considered to be responsible for the improved toughness. Their mechanical properties are reported in Table 8.

**Table 7:** **Blends with biodegradable elastomers and rubbers**.

**Elastomer category**	**Elastomer name**	**PLA/Elastomer (wt/wt)**	**Tensile strength (MPa)**	**Tensile modulus E (GPa)**	**Tensile elongation ε(%)**	**Izod impact strength (J/m)**	**References**
–	–	100/0	65	–	4	64 (Unnotched) 27 (Notched)	Li and Shimizu, 2007
Biodegradable PU Elastomer	Thermoplastic Poly(ether)urethane	70/30	31.5	–	363	315 (Unnotched)	
	Pellethane™2102-75A	70/30	–	–	410	769 (Notched)	Natureworks LLC, 2007
–	–	100/0	46.8	1.8	5.1	–	Zhang et al., 2009b
Biodegradable Polyamide Elastomer	Thermoplastic PAE	5	48.1	1.5	161.5	–	
		20	23.4	-	184.6	–	

**Table 8:** **Mechanical properties of different PLA blends with biodegradable flexible/soft polymers**.

**Formulation (wt.%)**	***E_t_* (MPa)**	**σ*_y_* (MPa)**	**ε*_b_*(%)**	**NIIE (KJ/m^2^)**			
**PLA/PHBV (Ma et al., 2013)**					**U-NIIE (KJ/m^2^)**	***E_f_*(GPa)**	**σ*_f_* (MPa)**
100/0		68	4	2.5	16	3.5	109
95/5		62	5	2.7	15	3.4	96
90/10		53	220	3.1	23	3.0	85
80/20		42	230	11	150n.f.	2.5	66
70/30		35	260	10	127n.f.	2.0	51
50/50		15	15	6	41	1.2	21
0/100		9	12	48	45n.f.	0.5	11
**PLA/PBAT (Jiang et al., 2006)**							
100/0	3400	63	3.7	2.6			
95/5	–	–	~115	–			
80/20	2600	47	>200	4.4			
**PLA/PEBA (Han et al., 2013)**							
100/0	1170 ± 42	60.0 ± 1.1	6.7 ± 0.4	4.5 ± 0.6			
95/5	1151 ± 75	49.3 ± 1.2	13.7 ± 0.8	7.1 ± 0.3			
90/10	1156 ± 44	46.8 ± 0.4	283 ± 18	7.4 ± 0.4			
85/15	1062 ± 48	42.5 ± 0.9	313 ± 2	9.1 ± 0.4			
80/20	1011 ± 41	42.4 ± 1.0	346 ± 18	39.3 ± 2.2			
70/30	911 ± 54	36.8 ± 0.6	335 ± 5	60.5 ± 1.0			
**PLA/PAE (Zhang et al., 2009b)**					***E_s_* (MPa)**	***T_g_*_PAE_ (°C)**	***T_g_*_PLA_ (°C)**
100/0	1814	46.8	5.1		2460		79.48
95/5	1517	48.1	161.5		2116	–47.31	77.85
90/10	1633	40.9	194.6		2017	–53.87	75.97
80/20	1240	23.7	184.6		1442	–57.89	74.47
70/30	1050	24.6	367.2		1395	–60.26	73.84
**PLA/NR (Bitinis et al., 2011)**							
Pristine PLA	2900 ± 100	63.1 ± 1.1	3.3 ± 0.4				
Processed PLA	3100 ± 40	58.0 ± 1.5	5.3 ± 0.7				
95/5	2500 ± 60	50.4 ± 1.6	48 ± 22				
90/10	2000 ± 50	40.1 ± 1.5	200 ± 14				
80/20	1800 ± 80	24.9 ± 0.9	73 ± 45				
**PLA/GMS (Ge et al., 2013)**							
100/0	1777 ± 42	69.8 ± 3.2	5.7 ± 0.3	4.7 ± 0.2			
95/5	1570 ± 44	44.8 ± 1.3	4.5 ± 0.5	8.1 ± 0.4			
90/10	1200 ± 12	41.9 ± 4.6	7.6 ± 2.4	8.5 ± 0.5			
85/15	1270 ± 36	39.7 ± 1.0	11 ± 5.0	15.5 ± 0.3			
80/20	1210 ± 17	35.1 ± 2.1	9.5 ± 6.5	36.7 ± 0.3			
75/25	1190 ± 24	32.4 ± 1.8	11 ± 3.1	46.1 ± 2.9			
70/30	695 ± 38	29.9 ± 2.6	45 ± 15.8	48.2 ± 4.6			

*E_t_, Tensile modulus; σ_y_, Tensile yield stress; ε_b_, Tensile elongation at break; NIE, Notched Izod Impact Energy; U-NIIE, Un-Notched Izod Impact Energy; E_f_, Flexural modulus; σ_f_, Flexural stress; E_s_, Storage modulus; T_g_, Glass transition temperature by DMA; n.f., Not (completely) fractured*.

Dynamic mechanical analysis (DMA) demonstrated a good compatibility between PAE and PLA blends. A gook tu od dispersion of PAE in PLA matrix was shown in SEM images. When the PAE content was fixed to 10%, the tensile strength of blend was similar to that of neat PLA, and the elongation increased significantly to 194.6%. Remarkably, the blends showed a wonderful shape-memory effect. PAE domains act as stress concentrators in system with the stress release locally and lead to energy-dissipation process. This prevents PLA matrix from breaking under high deformation, and lead to the PLA molecular orientation. Consequently, the blends submitted to deformation upon tensile load, and heating up the material reform the shape back to the original shape. Table 8 lists the obtained results.

#### Natural rubber

Natural rubber (NR) can be a good impact modifier candidate for PLA because it is derived from renewable resource. However, because of its incompatibility with PLA, it does not provide the desired improvement of PLA toughness. It has also several properties and appearance issues. Interestingly, epoxidized natural rubber (ENR) is more compatible with PLA. The toughness of the final material is dependent on the level of epoxy functions present in the ENR. Bitinis et al. investigated some formulations of natural rubber (NR)–PLA blends (Bitinis et al., 2011). The rubber phase was uniformly dispersed in the continuous PLA matrix with a droplet size ranging from 1.1 to 2.0 μm. The ductility of PLA was significantly improved from 5% for neat PLA to 200% by adding 10 wt.% NR as reported in Table 8. At this concentration, the rubber droplets provided an optimum balance between their coalescence and their beneficial effect provided on the material's physical and mechanical behavior, without sacrificing totally the transparency of the material. Moreover, the small molecules contained in the elastomeric phase could be acting as nucleating agent, favoring the crystallization ability of PLA. According to the authors, these materials are, therefore, very promising for industrial applications.

Zhang et al. toughened PLA with epoxidized natural rubber (ENR) by melt-blending in an internal mixer (Zhang et al., 2013a). Whilst the stiffness of the material was slightly reduced, the impact strength of the latter could be improved to 6-fold values as compared to that of pure PLA. Again, the authors attributed this enhancement to a good interfacial adhesion between ENR and PLA.

**Figure 8:**
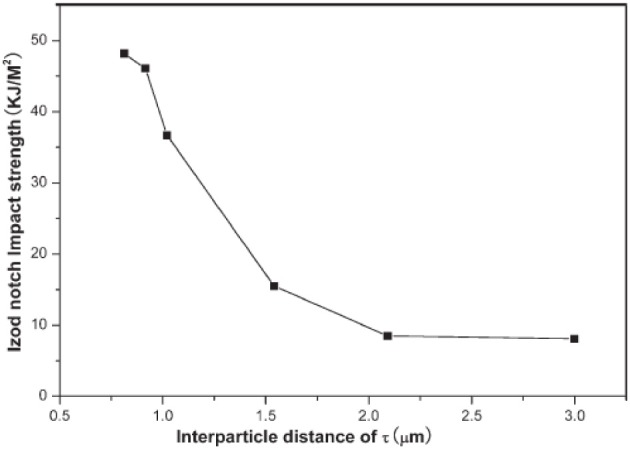
**The impact strength as a function of inter-particle distance of GMS**. Copyright (2012) Wiley; used with permission from Ge et al. (2013).

Ge et al. blended PLA with glycerol monostearate (GMS) (Ge et al., 2013) (Figure 8). SEM micrographs of the impact fracture surfaces of PLA/GMS blends had a relatively good separation and this phenomenon was in good agreement with their higher impact strength. The result showed that the addition of GMS enhanced the flexibility of PLA/GMS blends as compared to neat PLA. The impact strength changed from 4.7 kJ/m^2^ for neat PLA to 48.2 kJ/m^2^ for 70/30 PLA/GMS blend (Table 8).

**Figure 9:**
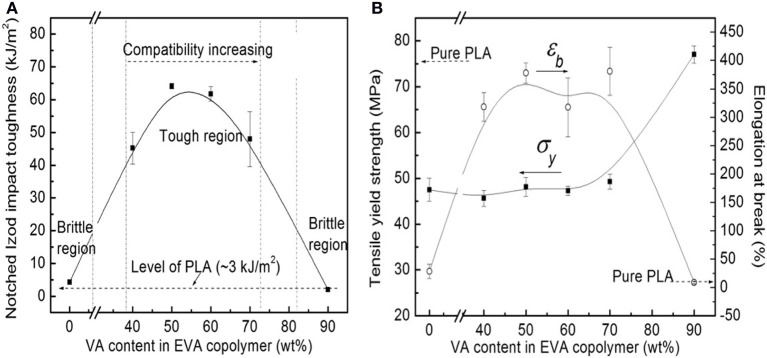
**(A)** Impact toughness and **(B)** tensile properties of the PLA/EVA (80/20) blends as a function of VA content in the EVA copolymers [Reprinted from Ma et al. (2012) with permission from Elseiver].

Ma et al. studied the influence of vinyl acetate (VA) content in ethylene-*co*-vinyl acetate copolymer (EVA) rubbers (Levapren®) on toughening mechanisms of PLA-based materials (Ma et al., 2012) (Figure 9). They showed that the increase of VA content improves the compatibility between the components of the blend, since PLA is miscible with PVAc (no phase separation). The toughness of the PLA/EVA (80/20 wt/wt) blends firstly increased with VA content up to 50 wt.% and then declined. At high VA content, it resulted the formation of small EVA particles that could not cavitate under impact testing, whereas at low VA content, large EVA particle size was achieved. However, in the latter, there had a weak interfacial adhesion, affecting the toughness of the PLA/EVA blends. As a result, the optimum toughening efficiency of EVA on PLA was obtained at VA content of 50–60 wt.%. The EVA with VA content of 50 wt.% (i.e., EVA50) was selected to study the toughening effect of EVA content in the PLA/EVA blends. Even 5 wt.% EVA50 could already make PLA ductile (ε_*b*_ ≈ 300%). However, the notched Izod impact toughness of this blend was not obviously improved due to a strain-rate dependence of the rubber cavitation (Dompas and Groeninckx, 1994; Jansen et al., 1999). Interestingly, the notched Izod impact toughness of the PLA/EVA50 blends was considerably improved in presence of 15 wt.% EVA50. By further increasing the EVA50 content, super-tough PLA/EVA50 blends could be obtained. The reason for brittleness of amorphous polymers is strain-localization, which could be delocalized by the dispersed rubber phase via a (pre)cavitation process. The morphology of PLA/EVA blends could be tuned by the VA content in the EVA copolymers as well as with the EVA content within the blends. The moderate particle size and the low modulus of the non-crosslinked EVA rubber particles are suitable for cavitation in the presence of tri-axial strain/stress [66, (Bucknall and Paul, 2009)]. Consequently, internal EVA rubber cavitation in the PLA matrix occurred under both the impact and tensile testing. Meanwhile, no obvious crazes were observed after deformation. In this regard, internal rubber cavitation in combination with matrix yielding is proposed to be the dominant toughening mechanism for the PLA/EVA blends.

To improve its toughness and crystallization, Zhang et al. (2013c) melt-blended PLA with ethylene/methyl acrylate/glycidyl methacrylate terpolymer (EGA) containing relatively high-concentration of epoxide groups (8 wt%). Although we cannot exclude any coupling reaction between epoxide groups and end-functionality (hydroxyl) from PLA chains, the addition of EGA accelerated the crystallization rate and increased the final crystallinity of PLA in the blends. Significant enhancements in both toughness and flexibility of PLA were achieved by the incorporation of 20–30 wt% EGA. The impact strength increased from 3 kJ/m^2^ of neat PLA to 60 kJ/m^2^ and the elongation at break increased from 5 to 232% (Table 11). The failure mode changed from brittle to ductile fracture of the blend. The phase separated morphology with relatively good interfacial adhesion played an important role in the improvement in crystallization and toughness of the blend.

**Figure 10:**
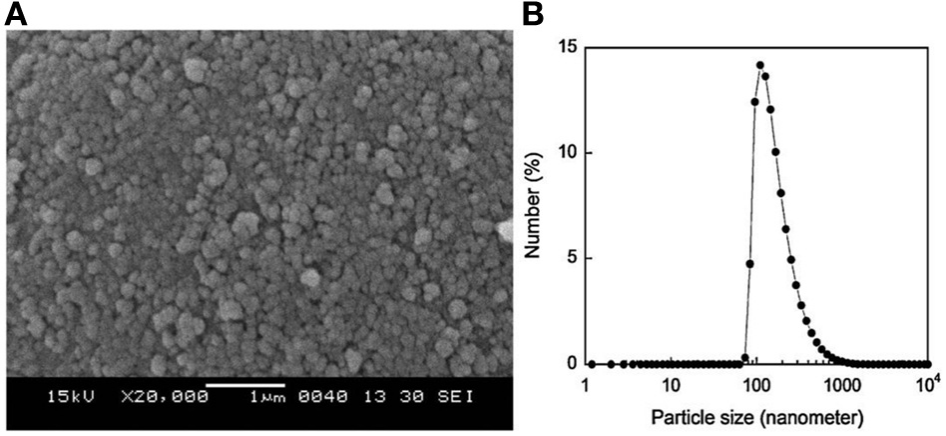
**(A)** SEM micrograph and **(B)** the article size distribution of the ultrafine acrylate rubber particles [Reprinted from Petchwattana et al. (2012) with permission from Elseiver].

Petchwattana et al. (2012) utilized ultrafine rubbery particles as toughening agent to reduce the brittleness of PLA. Elastomeric particles of acrylate rubber were added to PLA in the range from 0.1 to 10 wt% (Figure 10). Maximum reduction of the flexural modulus and the tensile modulus was achieved by 20 and 45% respectively, when the acrylate rubber content was of 10 wt%. However, under stress, the rubber-modified PLA could be uniaxially deformed to elongation at break of nearly 200%, accounting for an increase by 50 times in comparison to PLA. The toughening efficiency of the ultrafine rubber particles was also reflected through the significant increase in the impact strength by a four-fold factor. Fractographs of the acrylate rubber-modified PLA revealed a plastic deformation and a good dispersion and adhesion of the rubber particles within the PLA matrix. Therefore, they played an important role in dissipating the energy by formation of multiple crazes. The crazing mechanism was found to be the major impact mechanism of the acrylate rubber modified PLA system.

Jiang et al. (2006) and Li and Shimizu (2007) attributed the toughening behavior of the PLA-based blends to debonding at the matrix/particle interface during deformation, which released the hydrostatic stress and facilitated shear yielding to occur.

**Figure 11:**
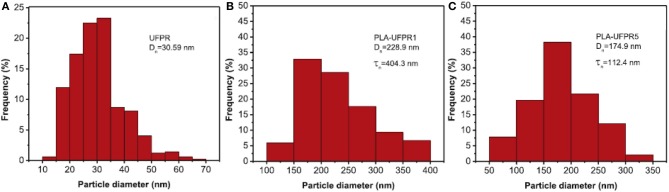
**Particle size distribution of UFPR (A), PLA-UFPR1 (B), and PLA-UFPR5 (C)**. D_n_, average particle diameter; sn, average matrix ligament thickness (interparticle distance). [Reprinted from Zhao et al. (2013) with permission from Elseiver].

Zhao et al. (2013) (Figure 11) used a unique ultrafine full-vulcanized powdered ethyl acrylate rubber (EA-UFPR) as toughening modifier for PLA. Largely improved tensile toughness was successfully achieved by the incorporation of only 1 wt% EA-UFPR, while the tensile strength and modulus of the blends were almost the same as pure PLA. The highly efficient toughening of UFPR on PLA could be mainly ascribed to the strong interfacial interaction between PLA and UFPR as well as a good dispersion of UFPR particles in PLA matrix. This induces de-bonding cavitation at the PLA/UFPR interfaces during stretching, leading to an extensive energy dissipation and superior tensile toughness. It should be highlighted that this work provided an effective toughening method to largely improve the mechanical properties of PLA without sacrificing its stiffness, which is very important for the wide application of PLA materials.

**Figure 12:**
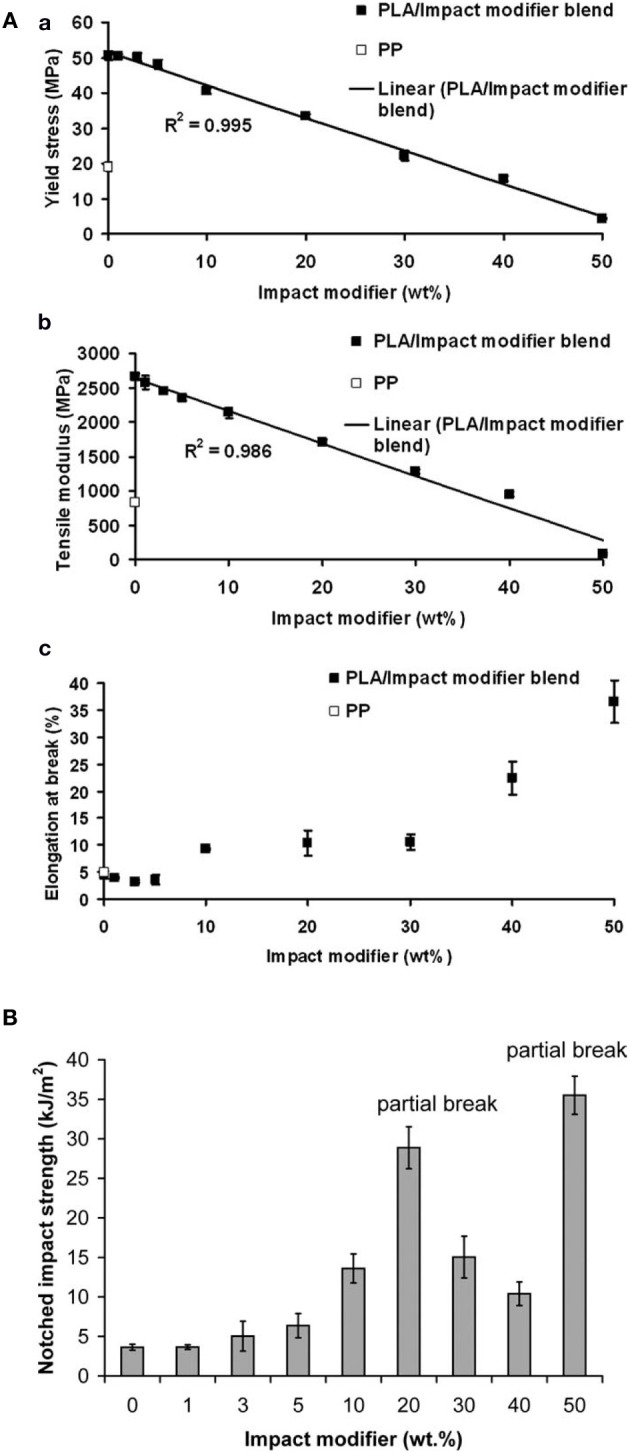
**(A)** Tensile properties of PLA and PLA/impact modifier blends. **(a)** Yield stress; **(b)** tensile modulus; and **(c)** elongation at break. **(B)** Notched impact strength of PLA and PLA/impact modifier blends. Notched impact strength for PP = 7.81 6 ± 1.50 kJ/m^2^. Copyright (2011) Wiley; used with permission from Taib et al. (2012).

Taib et al. (2012) toughened PLA with a commercially available ethylene acrylate copolymer impact modifier. PLA/impact modifier blends were partially miscible as confirmed by dynamic mechanical analysis. With increasing the impact modifier content, the stress-strain curves showed that the brittle behavior of PLA changed to ductile-failure. The blends showed some improvement in the elongation at break and notched impact strength, highlighting the toughening effects provided by the impact modifier again. In contrast, the yield stress and tensile modulus decreased with the increase in the impact modifier content (Figure 12A). Scanning electron microscopy micrographs revealed that the impact mechanisms among others involved shear-yielding or plastic deformation of the PLA matrix induced by interfacial de-bonding between the PLA and the impact modifier domains. In addition to shear-yielding of PLA, extensive deformation of the impact modifier domains was observed on the fractured surface, which accounts for the “partial” break of the blend after the impact test (Figure 12B).

#### PLA with polyethylene using PLLA-b-PE diblock copolymers as a compatibilizer (Anderson et al., 2003; Anderson and Hillmyer, 2004)

**Figure 13:**
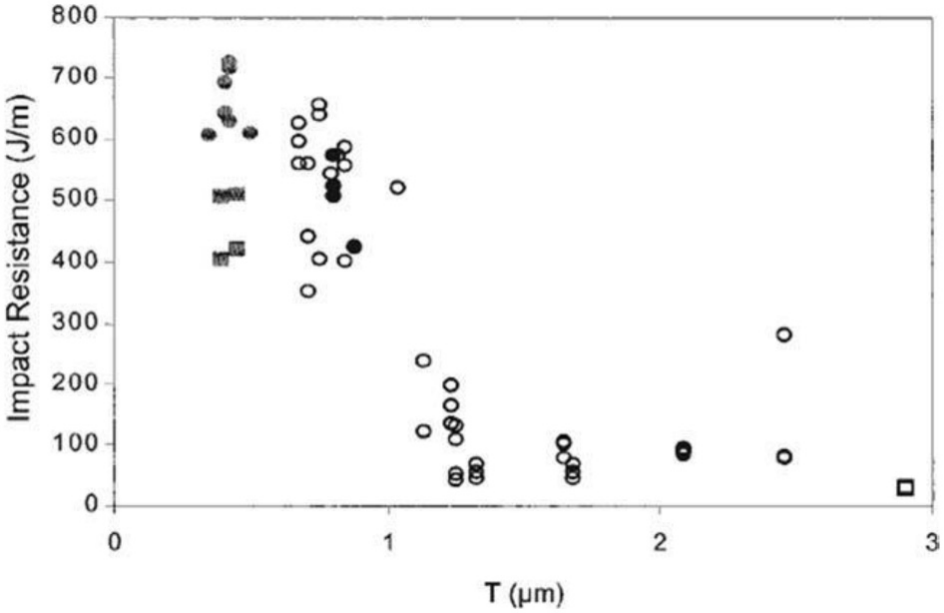
**Relationship between matrix ligament thickness (MLT) and impact resistance for: 80:20 PLLA/LLDPE binary blend (open circles); 80:20:5 PLLA/LLDPE/PLLA–PE(5–30) (black circles); 80:20:5 PLLA/LLDPE/PLLA-b-PE(30–30) (gray circles); 80:20 (w/w) PLA/LLDPE binary blend (open squares); and 80:20:5 (w/w) PLA/LLDPE/PLLA-b-PE(30–30) (gray squares)**. Copyright (2003) Wiley; used with permission from Anderson et al. (2003).

The addition of PLLA-b-PE block copolymers into the binary blend PLA/LLDPE resulted in improved interfacial adhesion and finer dispersion of LLDPE in PLA matrix. With the addition to the blend PLA/LLDPE (80/20, w/w) of 5 wt% of the block copolymer [with molecular weights for the PLA block above its entanglement molecular weight M_*c*_, *that is*, PLLA-*b*-PE (30–30 w/w)], the impact strength was drastically increased to 460 J/m (Figure 13). This difference was attributed to the superior ability of the block copolymer from the long PLLA block to suppress the coalescence of dispersed phase. Table 9 lists the impact strength properties as a function of the blend composition as well as some explanations of the occurring phenomena.

**Table 9:** **Results summary and explanation**.

**Blend system**	**Compatibilizer (PLLA-b-PE)**	**Impact strength (J/m)**	**Explanation**
a-PLA	–	12	
a-PLA/LLDPE (80wt%/20wt%)	–	34	
a-PLA/LLDPE (80wt%/20wt%)	5 wt%^a^	36	The difference is attributed to superior ability of the PLLA-b-PE from the longer PLLA block to suppress the coalescence of the dispersed phase
a-PLA/LLDPE (80wt%/20wt%)	5 wt%^b^	460	
PLLA	–	20	
PLLA/LLDPE (80wt%/20wt%)	–	350	Adhesion test showed a superior interfacial adhesion when used semicrystalline PLLA instead of amorphous a-PLA
PLLA/LLDPE (80wt%/20wt%)	5 wt%^a^	510	The tacticity effects on either the Mc of PLA or the miscibility degree of PLA matrix with LLDPE phase accounted for the difference between the two binary blends
PLLA/LLDPE (80wt%/20wt%)	5 wt%^b^	660	

*a-PLA, amorphous PLA; PLLA, semicrystalline PLA; LLDPE, linear low density polyethylene*.

a *The molecular weight of PLLA block in PLLA-b-PE is 5 kg/mol < M_c_ = 9 kg/mol*.

b *The molecular weight of PLLA block in PLLA-b-PE is 30 kg/mol > M_c_ = 9 kg/mol*.

*M_c_ = 9 kg/mol is the critical entanglement molecular weight of PLLA block in PLLA-b-PE*.

**Figure 14:**
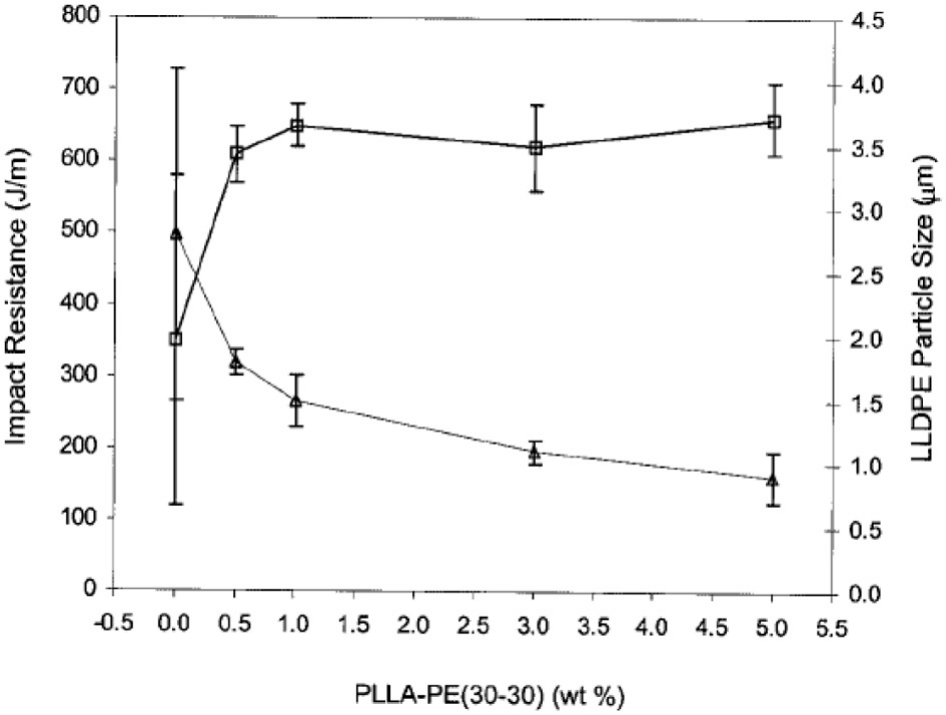
**Effect of the amount of PLLA–PE(30–30) block copolymer on the impact resistance (squares) and the LLDPE particle size (triangles) of 80: 20 PLLA/LLDPE blends**. Copyright (2003) Wiley; used with permission from Anderson et al. (2003).

By increasing the amounts of PLLA-b-PE (30–30 wt/wt) block copolymer in the PLLA/LLDPE (80/20, wt/wt) blends, the size of dispersed LLDPE particles was gradually reduced. At 3 wt.% of block copolymer, the size of the dispersed LLDPE particles began leveling off at less than 1.0 μm, and the impact resistance drastically increased (Figure 14 and Table 10).

**Table 10:** **Particle size analysis and impact resistance of PLLA homopolymer and blends [Copyright (2003) Wiley; used with permission from Anderson et al. (2003)]**.

**PLLA/LLDPE/PLLA-PE block**	**PLLA-PE block**	**LLDPE particle size (μm)**	**Izod impact resistance (J/m)**
100/0/0			20 ± 2
80/20/0		2.8 ± 1.3	350 ± 230
80/20/5	5–30	1.9 ± 0.2	510 ± 60
80/20/5	30–30	0.9 ± 0.2	660 ± 50

Meng et al. successfully synthetized poly(butyl acrylate) (PBA) in order to melt-blend with PLA using a Haake Rheometer (Meng et al., 2012). Dynamic rheology, SEM and DSC results showed that PLA was partially miscible with PBA. The crystallinity of PLA increased with the content of PBA (<15 wt.%). By increasing PBA content, the tensile strength and modulus of the blend decreased slightly, while the elongation at break and toughness dramatically increased (Table 11). The failure mode changes from brittle to ductile fracture of the blend with PBA as well. SEM micrographs revealed that a de-bonding-initiated shear yielding mechanism is involved in the toughening of the blend. Rheological investigation revealed that a phase segregation occurred at loading above 11 wt.% PBA. UV–vis light transmittance showed that PLA/PBA blends had a high transparency, but the transparency slightly decreased with the amount of PBA.

**Table 11:** **Mechanical properties of different PLA blends with non-biodegradable flexible/soft polymers**.

**Formulation (wt.%)**	***E_t_* (MPa)**	**σ*_y_* (MPa)**	**ε*_b_*(%)**	**NIIE (KJ/m^2^)**			
**PLA/EVA50 (Ma et al., 2012)**					**Hardness (Shore D)**	**E*_f_*(GPa)**	**σ*_f_* (MPa)**
100/0		75	9	3	86	3.7	105
95/5		68	310	2	85	3.3	90
90/10		61	390	5	84	2.9	75
85/15		54	430	32	82	2.7	70
80/20		45	340	64	80	2.4	65
70/30		37	400	83	76	1.9	50
**PLA/EGA (Zhang et al., 2013c)**							
100/0	1745 ± 39	60.0 ± 3.0	4.9 ± 0.3	3.0 ± 0.4			
90/10	1530 ± 35	44.3 ± 2.1	23.4 ± 3.6	3.9 ± 0.3			
80/20	1154 ± 42	33.8 ± 2.4	232.0 ± 26	59.8 ± 5.1			
70/30	945 ± 49	24.9 ± 1.3	126.0 ± 21	53.2 ± 8.4			
**PLA/PEBA (Petchwattana et al., 2012)**				**NIIE (J/m)**			
100/0	2750 ± 120	61.22 ± 1.42	3.46 ± 1.42	23.66 ± 1.33			
99.9/0.1	2660 ± 60	61.69 ± 1.99	3.53 ± 0.19	26.62 ± 1.87			
99.7/0.3	2680 ± 80	61.57 ± 1.76	5.01 ± 0.34	33.18 ± 2.01			
99.5/0.5	2550 ± 70	58.34 ± 0.94	8.94 ± 1.82	36.89 ± 2.43			
99.3/0.7	2310 ± 140	58.17 ± 1.83	15.1 ± 2.07	38.12 ± 1.95			
99/1	2350 ± 380	58.69 ± 0.91	19.8 ± 4.97	52.15 ± 2.57			
97/3	2050 ± 120	53.89 ± 0.84	53.7 ± 4.93	64.59 ± 3.46			
95/5	2150 ± 220	54.22 ± 0.97	124 ± 25.9	86.95 ± 4.65			
93/7	2040 ± 210	50.31 ± 0.93	167 ± 24.4	96.21 ± 4.99			
90/10	2000 ± 250	48.98 ± 1.79	198 ± 31.7	101.0 ± 5.63			
**PLA/UFPR (Zhao et al., 2013)**							
100/0	2062 ± 12	68.05 ± 1.42	6.08 ± 0.36	1.60 ± 0.21			
99.5/0.5	1922 ± 66	67.53 ± 1.99	106.60 ± 15.08	2.00 ± 0.15			
99/1	1896 ± 2	66.26 ± 1.76	219.93 ± 2.64	2.2 ± 0.23			
97/3	1768 ± 54	65.67 ± 0.94	231.45 ± 20.55	2.6 ± 0.37			
95/5	2029 ± 129	65.39 ± 1.83	215.63 ± 12.21	3.20 ± 0.19			
**PLA/PBA (Meng et al., 2012)**					**Tensile toughness^a^ (MJ/m^3^)**		
100/0	3510	68	4.52		2.13		
95/5	1540	51.77	31.52		3.7		
92/8	1490	44.79	74.62		17.0		
89/11	1440	40.82	173.98		41.74		
85/15	1330	41.01	174.52		47.02		

*E_t_, Tensile modulus; σ_y_, Tensile yield stress; ε_b_, Tensile elongation at break; NIIE, Notched Izod Impact Energy; E_f_, Flexural modulus; σ_f_, Flexural stress*.

a *Calculated as the area under stress-strain curve*.

***Commercially available impact modifiers for PLA.*** Recently, several polymeric impact modifiers have been specifically produced and commercialized in order to toughen brittle PLA (Table 12). These impact modifiers may be based on either linear thermoplastics/elastomers having a low glass transition temperature or crosslinked core-shell block copolymers, where the core is mainly a rubbery soft block encapsulated by a glassy and rigid shell that brings a good interfacial compatibilization with the matrix. In the optimal conditions (dispersion, compatibilization/adhesion, size and size distribution…), they dissipate the mechanical energy, retarding the initiation and propagation of micro-cracks through the polymer matrix.

**Table 12:** **Injection moulded properties of PLA containing various commercial impact modifiers**.

**Manufacturer**	**Impact modifier (IM)**	**Nature**	**Characteristics of PLA/IM**	**Optimal load in PLA**	**IM loading (%)**	**Notched izod impact (J/m)**	**Tensile yield (MPa)**	**Elongation^a^ (%)**
Sukano	Sukano® PLA im S550 (Sukano Co., 2008; Scaffaro et al., 2011)	Linear elastomer	Transparent, compostable, cost effective, immiscible	4% (impact resistance improved by a factor of 10)	8	141 (87)		8 (3)
	Sukano® PLA im S555 (Co, 2010)	Linear elastomer	Transparent, compostable, cost effective					
PolyOne	OnCap™ BIO Impact T (http://www.polyone.com/en-us/docs/Documents/OnCap%20BIO%20Impact%20T.pdf. Accessed on 2008.; http://www.polyone.com/en-us/news/Press%20Release%20Attachments/Chinaplas%20OnCap%20BIO%20impact%20T.pdf. Accessed on April 19, 2010.; Scaffaro et al., 2011)	Linear elastomer	Transparent, tear resistant, immiscible		12.5	124 (87)		8 (3)
DuPont	Biomax® Strong 100 (DuPont Co., 2010).	Ethylene-acrylate copolymer	Non-food applications	<5% for good toughness and clarity	12			255
	Biomax® Strong 120	Ethylene-acrylate copolymer	Food packaging					
	Hytrel™3078	Aliphatic/aromatic copolyester	Very compatible	5–10% (High elongation at break and impact properties)	30	198 (27)	34 (62)	430 (10)
Rohm and Hass	Polaroid™ BPM-500	Acrylic-based	Transparent	3–5% (dart drop impact increased by four time with respect to neat PLA)				
	Polaroid™ KM 334				15	59 (27)	37 (62)	165 (10)
	Polaroid™ BTA 753				20	112 (27)	36 (62)	300 (10)
	Polaroid™ EXL 3691A				20	64 (27)	43 (62)	280 (10)
	Polaroid™ EXL 2314				20	53 (27)	38 (62)	250 (10)
Dow chemical company	Polaroid™ BPM-515	Acrylic-based	Transparent	1% (higher efficiency than Polaroid™ BPM-500)				
	Pellethane™2102-75A				30	769 (27)	42 (62)	410 (10)
Arkema	Biostrength™ 130	Acrylic core-shell structure	Transparent	2–6 wt.%				
	Biostrength™ 150	MMA-butadiene-styrene core-shell structure	Opaque	2–6 wt.%				
	Biostrength™ 280	Acrylic core-shell structure	Transparent	2–6 wt.%				
Procter and gamble metabolix	Nodax™	Aliphatic copolyester (PHA)		10–20 wt.% (rubber domains size 0.2–1.0 μm)				
Karton polymers LLC.	Karton™FG 1901X	Functionalized Elastomer			20	-	33 (62)	100 (10)
BASF	Ecoflex™	Aliphatic/aromatic copolyester	Very compatible	5–10% (High elongation at break and impact properties	30	128 (27)	43 (62)	100 (10)
Showa higher polymer	Bionelle™3001	Aliphatic polyester			15	48 (27)	59 (62)	230 (10)
Crompton corporation	Blendex™ 415				15	48 (27)	44 (62)	230 (10)
	Blendex™ 360				20	107 (27)	47 (62)	280 (10)
	Blendex™ 338	ABS resin (70% of butadiene)			20	518 (27)	43 (62)	281 (10)
Reference	PLA				0			10

a *According to ASTM D-638 at 2.0 inch/min*.

Recently, Scaffaro et al. (2011) have compared toughening effects of OnCap™ BIO Impact T and Sukano® PLA im S550 on PLA. Both modifiers were immiscible with PLA, but Sukano® PLA im S550 displayed a more homogeneous dispersion in the PLA matrix. It was found that none of the impact modifiers brought obvious increase in elongation to PLA. The maximum Izod impact strength of 141 J/m was achieved by adding 8 wt.% Sukano® PLA im S550, while the impact strength increased only to 124 J/m even with the addition of OnCap™ BIO Impact T. Murariu et al. (2008b) studied toughening effects of Biomax Strong® 100 on PLA and high-filled PLA/b-calcium sulphate anhydrite (AII) composites. Notched Izod impact strength of PLA containing 5 and 10 wt.% Biomax Strong® 100 increased from 2.6 kJ/m^2^ of the neat PLA to 4.6 and 12.4 kJ/m^2^, respectively. Elongation at break was more than 25% for the blend containing 10 wt.% of the impact modifier, while tensile strength and modulus of PLA gradually decreased with the addition of the impact modifier. Addition of 5 and 10 wt% of the impact modifier to the PLA/AII (70/30, wt/wt) composite also increased their impact strength to 4.5 and 5.7 kJ/m^2^, respectively. Impact strength slightly decreased with further increase of the filler loading to 40 wt.%, but remained higher than that of both the unmodified composites and the neat PLA. On the other hand, for the PLA composites containing 40% of filler, tensile strength and elongation markedly decreased with the incorporation of the impact modifier. Zhu et al. (2009) studied the films of PLA blends containing either Biomax Strong® 100 or Sukano® PLA im S550 as a toughening agent. It was shown that the modulus decreased when increasing the concentration of Biomax Strong® 100 modifier, but was relatively independent of the concentration of Sukano® PLA im S550. The maximum elongation was of 255% in presence of 12 wt.% of BiomaxVR Strong 100 and of 240% in presence of 8 wt.% of Sukano® PLA im S550, while elongation at break of neat PLA was of about 90%. For a given composition, the latter impact modifier gave a clearer film than Biomax® Strong 100, but the clarity of films decreased as the concentration increased for both toughening agents. Afrifah and Matuana (2010) investigated the toughening mechanisms of PLA blended with an ethylene/acrylate copolymer (EAC) to show a mode of fracture through crazing or microcracking and debonding of impact modifier particles with the matrix. This resulted in brittle failure at low content. Higher impact modifier content than 10 wt% revealed fracture mechanisms including impact modifier debonding, fibrillation, crack bridging, and matrix shear yielding, resulting in a ductile behavior. They also demonstrated that Biomax® Strong 100 yielded superior toughening on semi-crystalline PLA over amorphous PLA. With 40 wt.% of the toughening agent, the notched Izod impact strength of the semi-crystalline PLA increased from 16.9 J/m for amorphous PLA to 248.4 J/m for semi-crystalline PLA. In addition, the presence of 15 wt.% Biomax® Strong 100 reduced the brittle-to-ductile transition temperature of PLA, as revealed by the notched Izod impact test data from the frozen specimens. Ito et al. (2010) investigated the fracture mechanism of neat PLA and PLA blends toughened with an acrylic core–shell modifier. The acrylic modifier was composed of a crosslinked alkyl acrylate rubber core and PMMA shell, and the particle size was in the range of 100–300 nm. Plane strain compression testing of PLA clearly showed strong softening after yielding. Because the stress for craze nucleation was close to that of yield stress, brittle fracture occurred for neat PLA. Addition of the acrylic modifier significantly lowered the yield stress and formed many microvoids. The release of strain constrained by microvoiding and the decrease of yield stress led to the relaxation of stress concentration, and therefore the toughness was improved moderately. Table 9 summarizes the reported mechanical properties of some of highly toughened PLA blends prepared *via* melt-blending. From Table 9, it results that in order for a rubbery polymer to impart toughness to PLA or any other polymer, several criteria must be encountered as follows (Natureworks LLC, 2007):

– the rubber must be distributed as small domains (usually 0.1–1.0 μm) in the matrix polymer;
– the rubber must have a good interfacial adhesion to PLA;
– the glass transition temperature of the rubber must be at least 20°C lower that the test/use temperature;
– the molecular weight of the rubber must not be too low;
– the rubber should not be miscible, to a certain extent, with the polymer matrix;
– and the rubber must be thermally stable to PLA processing temperatures.

These factors will allow the rubber to induce energy dissipation mechanisms in PLA, which retard crack-initiation and propagation and ultimately result in a material with improved toughness. PLA is similar to many polymers that can undergo plastic flow mechanisms, initiated by dispersed rubber domains. The increase in toughness comes from the transfer of the impact energy to plastic flow, either in the form of crazing or shear yielding mechanisms through a large volume fraction of polymer. In PLA, excellent toughness balance can be obtained with 15–25% of impact modifier, even if little improvement can be seen by the addition of 3–5% of impact modifier. Typically like most conventional thermoplastics, PLA can be toughened after blending with rubbery polymers such as low modulus polyesters, linear elastomers, or cross-linked core-shell impact modifiers, which have been observed to impart the highest degree of toughening in PLA. These modifiers typically consist of a low *T_g_* crosslinked rubbery core (*T_g_* < −10°C) encapsulated by a glassy shell polymer (*T_g_* > 50°C) that has good interfacial adhesion with the matrix polymer. When well-dispersed, these modifiers act as nanoscale or microscale rubbery domains that dissipate mechanical energy to retard or arrest crack initiation and propagation through the polymer. Some possible major drawbacks resulting from blending PLA with impact modifiers are the dispersion of the micro-domains in the PLA matrix and the transparency of the resulting material. The latter case depends to some extent on the dispersion of the micro-domains and their size. The addition of impact modifier to PLA often results in a substantial decrease in clarity of the toughened blend, although high clarity is required for many PLA applications. To retain the good clarity and transparency of PLA, for instance, small dispersed particles have to have a similar refractive index as PLA as well as the particle size has to be inferior to the visible light wavelength. The use of very small rubber particles with refractive indexes comparable to that of PLA can help maintaining transparency (refractive index in the range of from 1.430 to 1.485). This can be achieved if the added rubber is slightly compatible with PLA. Moreover, a poor compatibility and interfacial adhesion can also result in partially dispersed and large cluster-like domains responsible for the de-bonding of the rubber phase, void-formation, and a premature failure in a brittle mode. The main negative consequence resulting from the incorporation of toughening agents into PLA is the reduction of the material stiffness (elastic modulus). Accordingly, many researchers have investigated the incorporation of rigid fillers in order to compensate the loss of stiffness.

Generally, fillers or fibers are incorporated in PLA to either reduce the cost or modify the physical, rheological, or optical properties of the polymer. Starch is for instance an excellent example, which is available at less than $ 0.10/pound and which retains both renewability and biodegradability characteristics of PLA while enhancing some structural and mechanical properties at room or elevated temperature. Some additives (e.g., talc), can increase the nucleation rates and crystallinity, and thereby improve heat resistance of PLA-based materials (Bopp and Whelan, 2003). Fillers and fibers can also increase the stiffness of PLA and, to some extent, enhance the toughness of PLA materials. This can be explained by the fact that the crystallization extent of PLA is enhanced on incorporation of fillers, and therefore yielding a ductile behavior for the resulting PLA-based materials (Urayama et al., 2003). In order to get the maximum benefits from the fibers or fillers, several factors must be considered. For instance, optimizing the extruder screw configuration, through-put rate, screw speed, temperature and other process parameters are necessary. The optimal particle size of the filler is generally in the range of 0.1–12 μm (Ikado et al., 1997). A good and uniform dispersion must be achieved as well. This is normally obtained by controlling the addition of the compatibilizers during processing (Kjeschke et al., 2001), which help the dispersion of filler/fiber as well as minimizes micro-defects in blends that can cause embrittlement. For instance, the affinity of organically modified clay in PLLA/PBS blends was found to be critical for the properties enhancement of resulting composites (Chen et al., 2005). When a commercially available nanoclays, i.e., 10 wt% Cloisite® 25A was used as compatibilizer into the PLLA/PBS (75/25, w/w) blend, the tensile modulus of blends increased from 1.08 GPa to 1.94 GPa, but the elongation at break was sacrificed from 71.8 to 3.6% at the same time, which was even lower than that of neat PLLA (6.9%). In contrast, using an epoxy-functionalized organoclay (TFC) at the same amounts that was able to react with the extremities (carboxylic/hydroxylic) of both polyesters, not only retained high tensile modulus, but also increased elongation to 118%. Similar compatibilizing effect of TFC on the PLA/PBSA (75/25 w/w) blends were reported (Chen and Yoon, 2005). Odent et al. reported immiscible polymer blends made of PLA toughened with Biomax Strong® 100 in order to elaborate ultratough PLA-based materials mediated with nanoparticles (ref. Odent et al., 2013c). The co-addition of 10 wt% of Biomax Strong® 100 and 10 wt% of silica nanoparticles (CAB-O-SIL TS-530 from CABOT) provided an increase from 2.7 kJ/m^2^ to 30.2 kJ/m^2^, which is related to the formation of peculiar morphologies (from round-like nodules to elongated structure) mediated by the localization of nanoparticles at the interface PLA/impact modifier. Same improvement was also reached by replacing silica with organomodified layered aluminosilicate (clay), with value of 32.6, 37.6 and 21.9 kJ/m^2^ with only 1 wt% of Cloisite 20A, Cloisite 25A, and Cloisite 30B, respectively. Coupling agents are often used with glass fibers (Mochizuki and Suzuki, 2004) or coated fillers to enhance the interfacial adhesion of the additive to the matrix polymer, more particularly when polar additives are combined with non-polar polymers. Silane and titinate coupling agents with various structures, which depends on the polymer to be blended, are often embedded onto glass fibers and inorganic particulate fillers. These coupling agents can have beneficial effects on dispersion, toughness and rheology, and often allow higher levels of incorporation. However, the desired beneficial effects from the addition of fillers and fibers can have some negative consequences. High levels of fillers/fibers can significantly increase viscosity, cause shear heating and degradation (molecular weight loss and color formation), and affect the ability to fill thin walled parts. In the case of natural fibers, they contain high levels of moisture, requiring to apply extensive drying step before processing. It is worth noting that adding large amounts of natural fiber into the extruder requires using side-feeders for uniform extrusion operations. The batch-to-batch variation in natural fiber composition and quality can lead to consistency problems in the final blend as well (http://www.natureworksllc.com/~/media/Technical_Resources/Properties_Documents/PropertiesDocument_Fillers-and-Fibers_pdf.pdf).

Visual problems are also an issue with flow lines, poor colorability, and opacity. Table 13 is listing the mechanical properties of some fillers blended with PLA.

**Table 13:** **Mechanical properties of some fillers blended with PLA (http://www.natureworksllc.com/~/media/Technical_Resources/Properties_Documents/PropertiesDocument_Fillers-and-Fibers_pdf.pdf)**.

**PLA filled with**	**Filler loading (%)**	**Flex modulus^a^ (MPa)**	**Dart impact at 23°C (KJ/m^2^)**	**IZOD impact^b^ (notched) (J/m)**	**IZOD impact^b^ (un-notched) (KJ/m^2^)**
Specialty minerals MTAGD609 Talc	1.5	3940	123	43	331
	10	5002	112	27	272
	30	9248	69	27	176
Vicron 15–15 CaCO_3_	1.5	3809	107	32	272
	10	4287	128	27	288
	30	5606	128	32	187
Specialty minerals Mica 5040	1.5	4009	123	32	256
	10	5366	139	27	198
	30	9874	85	32	123
Synthetic silicate	1.5	3855	144	32	304
	10	4345	117	27	214
	30	5761	96	21	112
Specialty minerals EMforce™ Bio	1.5	3876	128	32	203
	10	4457	134	32	171
	30	5687	1057	123	294
Unmodified NatureWorkx™ PLA 4032D		3651	160	37	235

a *ASTM D 790*.

b *ASTM D 256-92*.

Jiang et al. (Long et al., 2009) compared the effects of organo-modified montmorillonite (OMMT) and nanosized precipitated calcium carbonate (NPCC) on the mechanical properties of PLA/PBAT/nanofiller ternary composites. Mechanical testing demonstrated that the composites containing OMMT exhibited higher tensile strength and Young's modulus, but with lower elongation, as compared to those containing NPCC. When 25 wt.% PLA was replaced by maleic anhydride grafted PLA (PLA-*g*-MA), the elongation of the ternary composites was substantially increased, possibly as a result of the improved dispersion of the nanoparticles and enhanced interfacial adhesion from maleic anhydride moieties along the PLA backbone. Among these composites, PLA/10 wt.% PBAT/2.5 wt.% OMMT/25 wt.% PLA-*g*-MA demonstrated the best overall properties with 87% retention of tensile strength of pure PLA, slightly higher modulus and significantly improved elongation at break (16.5 times than that of neat PLA). Teamsinsungvon et al. (2010) also reinforced PLA/PBAT blends using microsized precipitated CaCO_3_ and achieved similar toughening effects on PLA/PBAT (80/20, wt/wt) blends. The incorporation of talc particles significantly accelerated the crystallization process of the PLA matrix (Battegazzore et al., 2011). The presence of crystals improved the thermo-mechanical properties. Talc provides both reinforcing and toughening effects on PLA (Yu et al., 2012). The reinforcing effect of talc particles can be mainly ascribed to the good interfacial adhesion between PLA matrix and the orientation of talc layers during processing. Interfacial debonding of PLA/talc composite can induce massive crazing, meanwhile talc particles diffused in PLA matrix can prevent from the void coalescence and propagation of the crazes, which increase the toughness of PLA. Additionally, talc layers aligned along the tensile direction make its toughening effect on PLA more significant in tensile test because they induce more advantageous shear yielding and prevent microcracks from propagation along fracture direction. Some aggregation of talc particles can appear in composites at higher talc content, which act as a stress concentration points or weak points and cause poor toughness of PLA/talc composites. Recently, NatureWorks® has succeeded to develop the Ingeo™ 3801X grade by co-adding impact modifier, crystal accelerant, reinforcing agent and nucleating agent into PLA. This PLA-based grade is a high heat and impact resistance material. Table 14 shows its composition.

**Table 14:** **Formulation of the Ingeo™ 3801X grade (http://www.biocom.iastate.edu/workshop/2010workshop/presentations/dan_sawyer.pdf)**.

**Material**	**Commercial name**	**Supplier**	**Chemical**	**Formula weight fraction**	**25°C density [g/ml]**
Matrix	Ingeo™ 3001D	NatureWorks LCC	PLA	0.711	1.24
Impact modifier	Biostrength® 150	Arkema Inc.	Proprietary core-shell copolymer	0.100	1.00
Crystal accelerant	Plasthall® DOA	The HallStar Company	Dioctyl adipate	0.090	0.98
Reinforcing agent	ULTRATALC® 609	Specialty Minerals Inc.	<0.9 μm particle 3MgO.4SiO_2_.2H_2_O	0.090	2.8
Nucleating agent	LAK-301	Takemoto Oil & Fat Co. LTD	Aromatic sulfonate derivative	0.009	1.00

The bio-content of this material is of 70%. It was designed for non-food, opaque, semi-durable, and non-compostable applications. Due to its high crystallinity and rapid crystallization rate, the resulting blend has good thermal and dimensional stability. It is designed to be processed at fast cycle times. Its mechanical properties are summarized in Table 15.

**Table 15:** **Thermal and mechanical properties of the Ingeo™ 3801X grade (http://www.biocom.iastate.edu/workshop/2010workshop/presentations/dan_sawyer.pdf)**.

**Mechanical properties**	**Value**	**ASTM standard**
Tensile Modulus (MPa)	2980	D638
Tensile yield strength (MPa)	25.9	D638
Tensile elongation at break (%)	8.1	D638
Notched Izod impact (J/m)	144	D256
HDT B at 0.45 MPa (°C)	65	E2092
HDT at 0.114 MPa (°C)	140	E2092

Physical compounding of low or high molecular weight compounds offers a convenient approach to modifying biopolymers. Whether a good compatibility/affinity is present between both partners, the resulting blends exhibit good properties, being intermediate from that of each polymeric partner. However, only few biopolymer pairs are miscible or even compatible with each other. Therefore, chemical routes such as chemical modification or reactive compatibilization are required. Reactive blending has proven to be a simple, economically viable, and reliable technology for designing complex nanostructured polymeric blends. In this part, it will be pointed out, by reviewing the recent advances, that reactive extrusion technology represents the most promising approaches to tune the stiffness-toughness balance of bio-sourced polymeric materials. Reactive extrusion enables to manufacture biodegradable polymers through different routes of reactive modification (polymerization, grafting, compatibilization, branching, functionalization,…) in a cost-effective polymer processing (Michaeli et al., 1993; Mani et al., 1999). Most of the researchers employ this technology for the reactive compatibilization of PLA with a rubbery modifier in order to impart toughness to PLA. In the review, the *in situ* reactive compatibilization is defined here as the melt-blending process in which two polymers containing mutually reactive functionalities react with each other at the interface to combine them during melt blending, generating *in situ* block or graft copolymer. The *in situ* generated block or graft copolymer will be then able to compatibilize the blend by reducing the interfacial tension and by improving the interfacial adhesion. This leads to a significantly improved dispersibility of the rubber into much smaller particles (Baker et al., 2001). Compatibility is defined in this context as the ability of the rubber modifier to finely disperse into the main PLA phase in order to form stable morphologies of fine rubber particle dispersions with reduced interfacial tension and improved adhesion to resist delamination. Early patent literature recognized the need for some types of grafting reactions or an associative interaction between the polymeric components of the blend to obtain sufficient compatibility for good impact modification (Kray and Bellet, 1968; Seddon et al., 1971; Murch, 1974; Mason and Tuller, 1983). Interfacial compatibilization and toughening is achieved through grafting copolymers generated *in situ* at the interface during the melt blending. There are four fundamental requirements to be addressed for the *in situ* reactive compatibilization in an extrusion device:

– Sufficient mixing to achieve the desired distribution and dispersion of one polymer into another polymer;
– The presence of reactive functionalities in each phase capable of reacting together across the interphase;
– Reaction to take place within the available residence time in the processing device;
– Formation of stable bonds during processing.

In the case of polymers with no reactive chemical function (such as polyolefins), peroxides are used to create free polymeric macroradicals in the blend. Like in some cases the compatibilization cannot be achieved through the direct reaction between the free polymeric macro-radicals, low molecular weight (macro)monomers, or a mixture of low molecular weight (macro)monomers have to be grafted on the free polymeric radicals in order to functionalize the polymeric chains. The role of these grafted chemical functions is to (1) stabilize the macro-radicals, and thereby avoid any undesirable free radical side-reactions by localizing the free radical reactions at the polymers' interface, and (2) interact at the interface between the polymer components of the blend for compatibilization. PLA has been blended and reactive compatibilized with several biodegradable and non-biodegradable polymer modifiers that will be discussed here. We have to mention that other researchers have attempted using reactive extrusion technique to *in situ* synthetize the toughening agent for PLA.

#### Biodegradable hyperbranched polymers (HBP)

**Figure 15:**
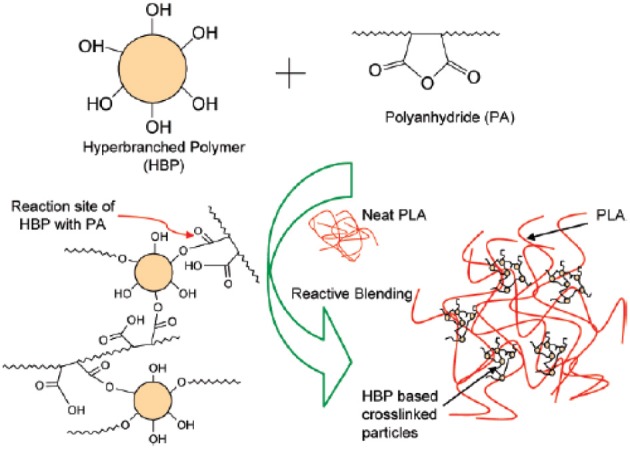
**Schematic illustrations of *in-situ* cross-linking of hyperbranched polymer (HBP) in the PLA melt with the help of a polyanhydride (PA)**. Adapted with permission from Bhardwaj and Mohanty (2007).

Hyperbranched polymer-based nanostructures (HBPs) have a globular molecular architecture with cavernous interiors, many peripheral functional end-groups, and low hydrodynamic volume and viscosity. They may have better miscibility with other polymers than the linear analogous (Seiler, 2002). Due to their inherently high surface area—volume ratio, structures engineered at the nano-meter length scales are increasingly played key-roles in the enhancement of the materials mechanical properties. In this regard, they have demonstrated a high potential to be used as impact modifiers for mechanical performances in a variety of industrial applications after a reactive process (Liu and Zhang, 2011). For instance, non-reactive melt-blending (physical compatibilization via H-bonding) of a hyperbranched biodegradable poly(ester amide) with PLA modestly enhance, yield strength. Moreover, the tensile failure mode changed from brittle to ductile fracture and led to a maximum tensile elongation at break of 50% compared to 3.7% for neat PLA using 20 wt.% HBP (Lin et al., 2007). This was explained by the fact that the dispersion of hyperbranched biodegradable poly(ester amide) was not fine enough to get its maximum benefits. In this respect, Bhardwaj and Mohanty (2007)proposed and demonstrated a new industrially relevant methodology to develop PLA-based materials, having outstanding stiffness-toughness balance through *in situ* cross-linking reactions. They *in-situ* cross-linked a hydroxyl functional hyperbranched polymer (HBP) with a polyanhydride (PA) in the PLA matrix during melt-processing (Figure 15). Transmission electron microscopy (TEM) and atomic force microscopy (AFM) revealed the sea-island morphology of PLA-cross-linked HBP reactive blend. The domain size of cross-linked HBP particles in the PLA matrix was less than 100 nm. Compared to unmodified neat PLA, the PLA/HBP/PA (92/5.4/2.6, wt/wt/wt) blend exhibited ~570% and ~847% improvement in the tensile toughness (17.4 MJ/m^3^ vs. 2.6 MJ/m^3^ for neat PLA) and elongation at break (48.3% vs. 5.1% for neat PLA), respectively. However, tensile modulus and strength of the blend slightly decreased from 3.6 GPa (neat PLA) to 2.8 GPa and from 76.6 MPa (neat PLA) to 63.9 MPa, respectively. The authors ascribed the increase in the ductility of modified PLA to the stress-whitening and the multiple crazing initiated in the presence of cross-linked HBP particles. As revealed by rheological data, the formation of a networked interface was associated with enhanced compatibility of the PLA-cross-linked HBP blend as compared to the PLA/HBP blend.

The effects on mechanical properties of hydroxyl-terminated hyperbranched poly(ester amide) (HBP) and isocyanate-terminated prepolymer of butadiene (ITPB), alone and in combination, were investigated with the aim to make tough PLA (Nyambo et al., 2012). The glass transition temperature did not change from that of neat PLA. Interestingly, due to synergistic effects, impact strength and elongation at break of the PLA/HBP/ITPB ternary blend were improved by over 86 and 100%, respectively. Physical and chemical interactions between the hydroxyl-terminated HBP and the ITPB (Scheme 2B) may be responsible for the synergistic effect on the improvements in impact strength without scarifying the tensile modulus and strength. Scanning electron microscopy (SEM) images on impact fractures showed evidence of stretched and course surface, which indicated a change in fracture behavior from brittle to ductile behavior after chemical modification. Accordingly, the impact strength of PLA can be easily enhanced using low additive loadings of 10 wt% *via* reactive extrusion with HBP and a suitable reactive compatibilizer such as ITPB. The modified PLA can address most issues related to neat brittle PLA, since it can exhibit a better stiffness–toughness balance and has the potential for use in durable commercial applications.

#### Soybean oil

PLLA/soybean oil binary blends containing unmodified soybean oil undergoes phase inversion at even low concentrations of soybean oil, leading to the release of the oil during blending. Therefore, the blends must be compatibilized (Chang et al., 2009). Ali et al. (2009) demonstrated that moderate improvements in the elongation at break of PLLA were gained by the addition of epoxidized soybean oil.

**Figure 16:**
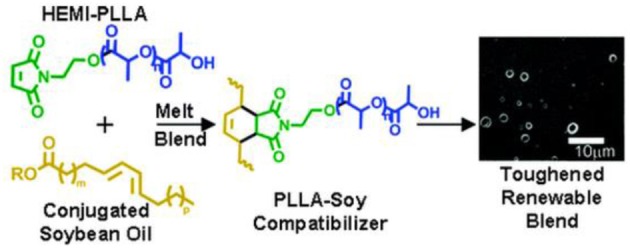
***In situ* Diels-Alder reaction coupling the two immiscible components HEMI-PLLA and conjugated soybean oil (CS) by means of reactive compatibilization**. Adapted with permission from Gramlich et al. (2010).

Robertson et al. (2010) explored how the polymerization and the optimization of soybean oil characteristics prior to blending improved its level of incorporation into PLLA and increased toughness compared to PLLA. They also demonstrated moderate improvements in the PLA/polysoybean oil blends regarding elongation at break and toughness of four and six times greater than those of unmodified PLLA, respectively. Gramlich et al. (2010) studied a more effective approach to toughen PLA consisting in the reactive compatibilization of conjugated soybean oil with PLLA. In a first step, bulk ring-opening polymerization via reactive extrusion (REx) of L-lactide using N-2-hydroxyethylmaleimide (HEMI) as a difunctional initiator and tin (II) 2-ethylhexanoate as a catalyst produced a high molecular weight reactive end-functionalized PLA (HEMI-PLLA). In a second step, REx of HEMI-PLLA and conjugated soybean oil (CS) was carried out through a Diels-Alder reaction in order to couple the two immiscible components *via* reactive compatibilization (Figure 16). Blends of HEMI-PLLA and 5 wt.% CS resulted in a greater than 17-fold increase in elongation to break compared to PLLA homopolymer and more than twice the elongation to break compared to a 5 wt.% CS blend with unreactive PLLA (Table 16). Analysis of the blend morphology indicated that the *in situ* formation of the compatibilizer at the HEMI-PLLA/CS interface decreased the CS droplet diameter to an optimal value (0.7 μm) compared to unreactive binary blends, explaining the toughening PLLA with CS.

**Table 16:** **Physical properties of melt blends of CS with PLLA-49 and HEMI-PLLA-67 (Adapted with permission from Gramlich et al. (2010)]**.

**Matrix polymer**	**W_CS0_ (%)^a^**	**W_CS_ (%)^b^**	**E (GPa)^c^**	**σ *_b_*(MPa)^d^**	**ε*_b_*(%)^e^**	***X_HP_* (%)^f^**	***X_CS_* (%)^g^**	***d_lm_* (μm)^h^**	**σ *_lm_*(μm)^i^**	**MLT (μm)^j^**
PLLA-49			2.4 ± 0.3	58 ± 3	5 ± 2					
HEMI-PLLA-67			3.0 ± 0.2	67 ± 9	4 ± 1					
PLLA-49	15	9	2.4 ± 0.3	28 ± 4	22 ± 7			1.81	1.8	3.1
HEMI-PLLA-67	15	7	2.0 ± 0.5	34 ± 2	50 ± 30	100	14	0.91	2.0	2.2
PLLA-49	5	4	2.1 ± 0.3	38 ± 1	30 ± 10			1.17	2.0	3.6
HEMI-PLLA-67	5	7	2.5 ± 0.2	37 ± 2	70 ± 30	98	44	0.70	2.1	2.0
PLLA-49/HEMI-PLLA-67^k^	5	6	2.3 ± 0.3	36 ± 3	20 ± 10	96	39	0.96	1.8	2.1
PLLA-49	2	2	2.6 ± 0.1	51 ± 1	5 ± 2			0.30	2.0	1.3
HEMI-PLLA-67	2	3	2.5 ± 0.2	54 ± 5	4 ± 2	66	70	0.35	1.3	0.5

a *Weight fraction of CS added to melt mixer*.

b *Weight fraction of CS incorporated into blends, by 1HNMR spectroscopy*.

c *Elastic modulus*.

d *Stress at break*.

e *Elongation to break*.

f *Conversion of HEMI end-groups for blends with HEMI-PLLA-67*.

g *Conversion of E,E isomers of CS added to mixer*.

h *Log-mean average CS droplet diameter*.

i *Log-mean CS droplet size distribution parameter*.

j *Matrix ligament thickness*.

k *Matrix polymer was a 50/50 blend of PLLA-49 and HEMI-PLLA-67*.

#### Use of glycidyl methacrylate (GMA) and its copolymers

**Scheme 3:**
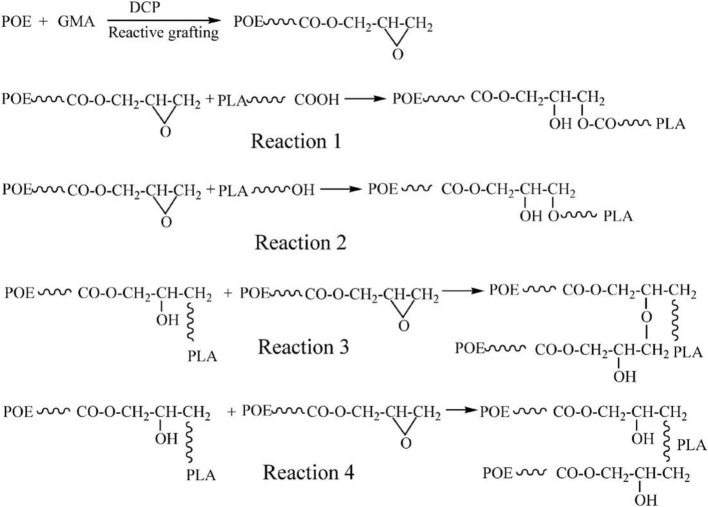
**The possible reactions in the melt reactive blends [Reprinted from Su et al. (2009) with permission from Elseiver]**.

**Figure 17:**
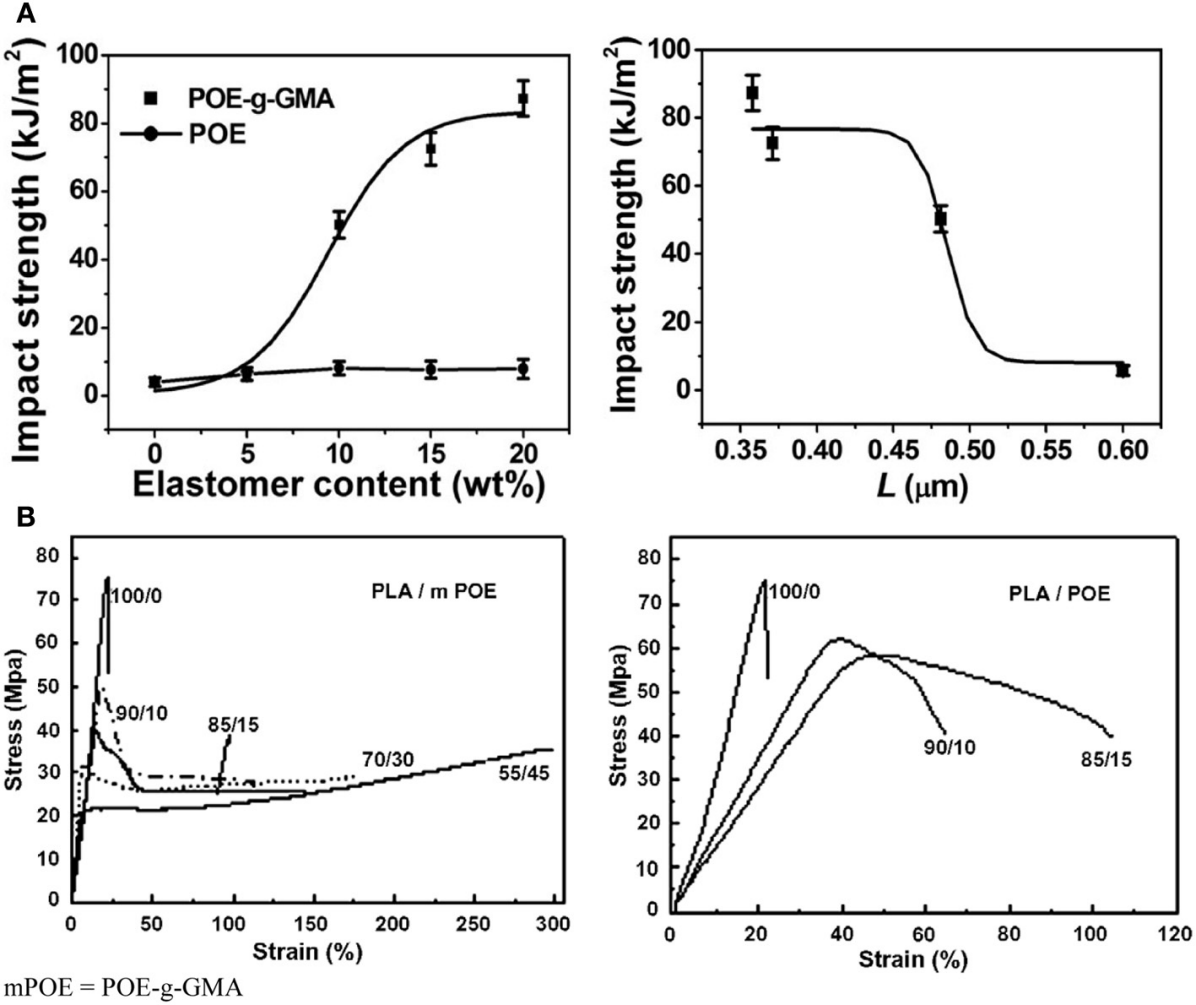
**(A)** Impact strength as a function of elastomer contents (to the left), and interparticle distance (to the right) for PLA/POE-g-GMA blends. Copyright (2012) Wiley; used with permission from Feng et al. (2013). **(B)** Strain–stress curve of PLA/POE and PLA/POE-g-GMA blends [Reprinted from Su et al. (2009) with permission from Elseiver].

The grafting effect on mechanical properties of poly(ethylene octene) (POE) with PLA via glycidyl methacrylate (GMA) was investigated (Su et al., 2009; Feng et al., 2013). POE-*g*-GMA was used to prepare high impact modified PLA/POE-*g*-GMA reactive blends (Scheme 3). The presence of GMA moieties enhanced the blends compatibility due to the coupling reactions between the carboxyl and hydroxyl end-groups from PLA and the epoxy groups from POE-*g*-GMA (Scheme 3). Moreover, morphology analysis demonstrated better wetting of the dispersed phase by the PLA matrix and finer dispersed particles by reactive blending. Accordingly, the effective interfacial compatibilization promoted by the grafting reaction was mainly responsible for the significant improvement of PLA toughening (Figures 17A,B). Interestingly, the highest toughening effect was obtained at lower particle size and interparticle distance, which were submicronic (Figure 17A).

**Scheme 4:**
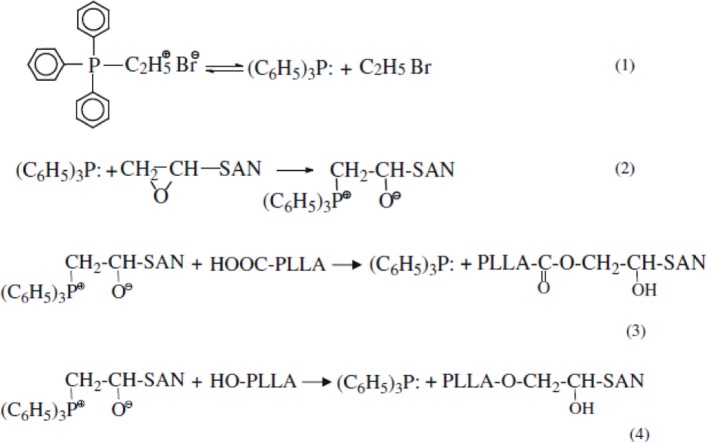
**Reaction of PLLA end groups with SAN-GMA under the catalyst of ETPB [Reprinted from Li and Shimizu (2009) with permission from Elseiver]**.

PLLA and acrylonitrile–butadiene–styrene copolymer (ABS) are thermodynamically immiscible and incompatible by simply melt blending them. Styrene acrylonitrile-glycidyl methacrylate copolymer (SAN-GMA) as a reactive compatibilizer and ethyltriphenyl phhosphonium bromide (ETPB) as a catalyst were thereby introduced during the reactive melt blending of PLA/ABS96 (Li and Shimizu, 2009). The epoxide group of SAN-GMA reacted with PLLA end-groups under the mixing conditions, and the addition of ETPB accelerated the reaction (Scheme 4). As a result, it was found that the size of the “salami-like” ABS domains in PLLA matrix significantly decreased and their dispersion improved by the addition of the reactive compatibilizer. A significant shift of glass transition temperatures for both PLLA and ABS indicated the improvement of the compatibility between PLLA and ABS. As a result, the compatibilized PLLA/ABS blends exhibited a very nice stiffness-toughness balance, i.e., an improvement of the impact strength and the elongation at break with a slight reduction in the modulus. For instance, the addition of 5 phr of SAN-GMA to the PLLA/ABS (70/30 wt/wt) blend increased elongation at break from 3.1 to 20.5% and impact strength from 63.8 to 81.1 kJ/m^2^. By further incorporating 0.02 phr ETPB, the elongation at break and impact strength of the blend increased to 23.8% and 123.9 kJ/m^2^, respectively.

**Figure 18:**
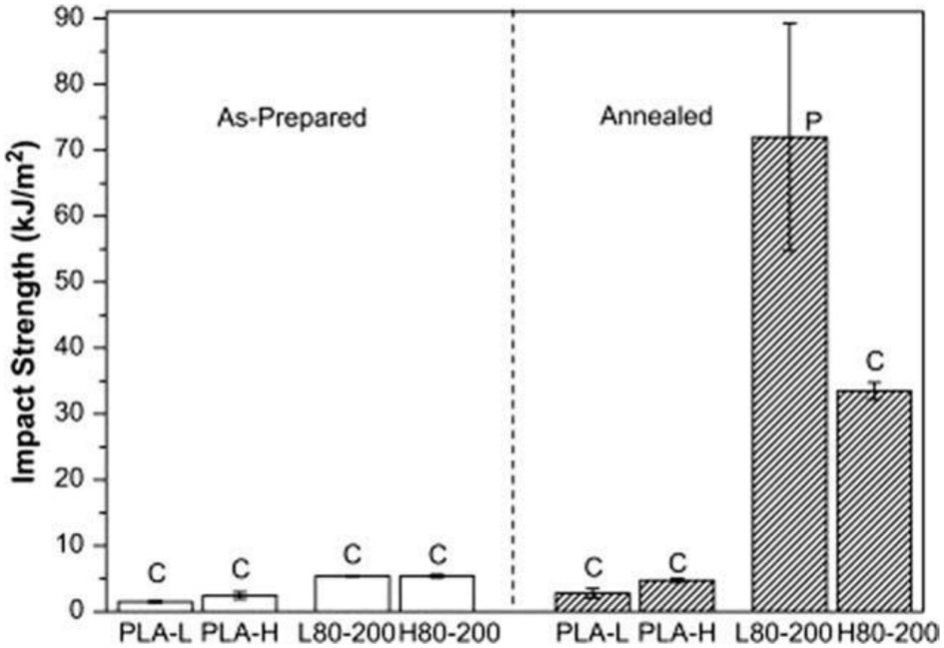
**Notched impact strength of PLAs and PLA/EGMA blends (C, complete break; P, partial break) [Oyama (2009) with permission from Elseiver]**.

Low and high molecular weight PLA (L-PLA and H-PLA, respectively) were blended with 20% of poly(ethylene-*co*-glycidyl methacrylate) (EGMA) (Oyama, 2009). The resulting blend had a high elongation above 200% compared to 5% for neat PLA. The notched Charpy impact was only 2 times that of neat PLA. After annealing, the injection-moulded specimens of the L-PLA/EGMA (80/20 wt/wt) blend at 90°C for 2.5 h showed that the impact strength significantly increased to 72 kJ/m^2^, about 50 times that of neat L-PLA. Moreover, the improvement in strength and modulus of the blend was accompanied by a significant decrease in elongation at break. With the higher molecular weight PLA (H-PLA) as matrix, such positive effect of annealing on impact strength appeared relatively less prominent (Figure 18). The author argued that the crystallization of the PLA matrix played a key-role in such significant improvement. It was demonstrated that the interfacial reaction (reactive compatibilization) between the polymeric components improved not only the dispersion of the second component but also the bonding between the particles and matrix to expect combination of crazing and shear yielding, contributing to the formation of the super-touch PLA materials, superior to commercially available acrylonitrile-butadiene-styrene (ABS) resins. Furthermore, these improvements in mechanical properties were achieved without scarifying the heat resistance of the material. The material highlights again the importance of interface control in the preparation of multicomponent materials.

**Scheme 5:**
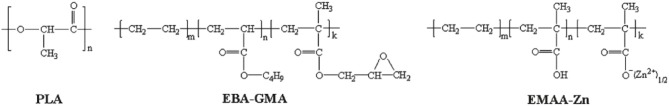
**Chemical structure of the three polymers used in the study**. Adapted with permission from Liu et al. (2011).

**Figure 19:**
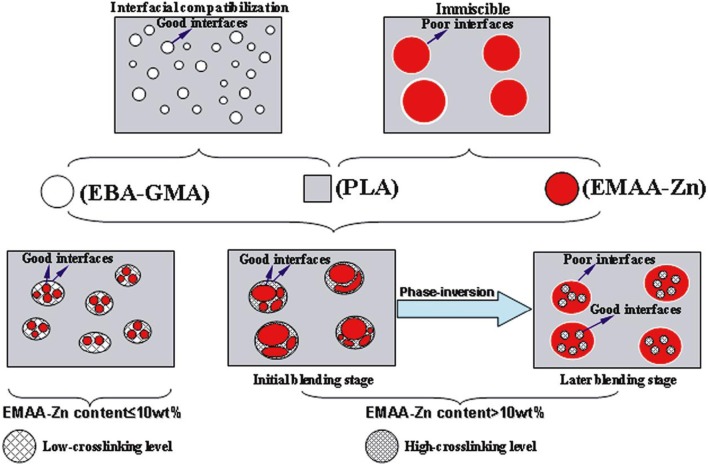
**Phase structure development for the studied PLA blend systems**. Adapted with permission from Liu et al. (2011).

Liu et al. (2010, 2011) and Song et al. (2012) studied extensively the reactive ternary blends of PLA with ethylene/n-butyl acrylate/glycidyl methacrylate (EBA-GMA) terpolymer and a zinc ionomer of ethylene/methacrylic acid (EMAA-Zn) using a Leistritz ZSE 18 twin-screw extruder having a L/D ratio of 40. The three polymeric components are represented in Scheme 5 and Figure 19.

**Figure 20:**
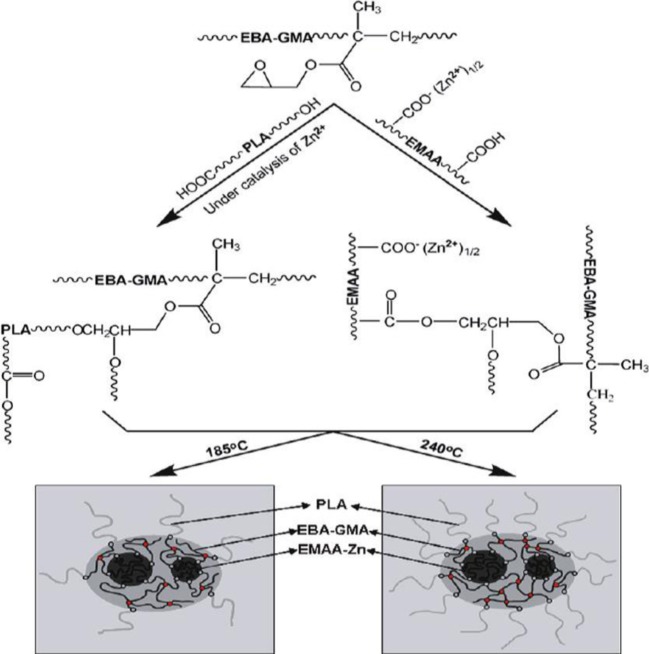
**Proposed reactions during the reactive blending process, together with schematic phase morphologies of the PLA/EBA-GMA/EMAA-Zn ternary blends extruded at 185 and 240°C respectively**. Adapted with permission from Liu et al. (2010).

The influence of the simultaneous dynamic vulcanization (crosslinking) and interfacial compatibilization and adhesion on mechanical and impact performance of the reactive PLA-based ternary blends was investigated. It was demonstrated that the EBA-GMA/EMMA-Zn ratio played a crucial role in determining the phase-morphology. Interestingly, the increase of the EMAA-Zn content gradually turned the phase of the latter from occluded sub-inclusions into a continuous phase within the “salami”-like micro-structure (domain-in-domain morphology) as revealed by TEM in the case of the ternary blends. It was reasonably proposed that when the EMAA-Zn content exceeded 10%, a phase inversion within the sub-structure of the dispersed phase domains could likely take place, which would account for the pronounced deterioration in interfacial wetting of the dispersed particles by the PLA matrix in these cases. The phase structure development for the studied PLA blend systems are schematized in Figure 20. The EMAA-Zn domains were finally occluded inside the EBA-GMA particles which were homogeneously dispersed in PLA.

Interestingly, it was demonstrated that at higher extrusion temperature (240 vs. 185°C), not only the carboxyl groups in the EMAA-Zn ionomer were able to trigger more cross-linking reactions *via* the epoxy groups in the EBA-GMA phase, but also more PLA macromolecules were grafted at the interface between PLA and the elastomer (Figure 20). The Zn ions further catalyzed the reactions. According to the SEM micrographs, this was confirmed by the better wetting of the dispersed phase by PLA matrix at higher blending temperature. Accordingly, effective interfacial compatibilization and adhesion were achieved at higher compounding temperature.

**Figure 21:**
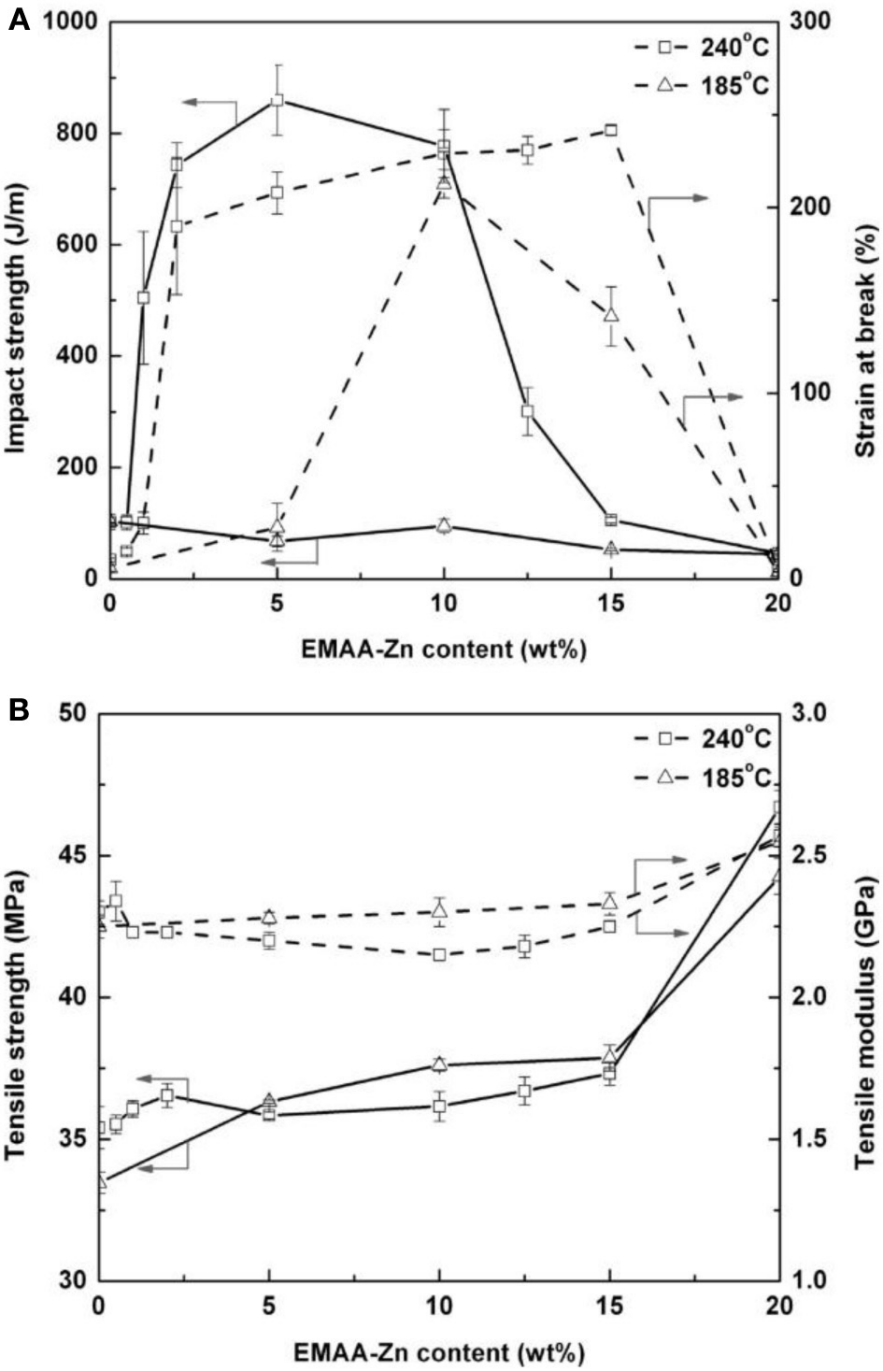
**Mechanical properties of PLA/EBA-GMA/EMAA-Zn (80/x/y in weight, x+ y = 20) blends as functions of weight content of added EMAA-Zn under 240°C vs. 185°C: (A) impact strength (solid line) and strain at break (%) (dashed line); (B) tensile strength (solid line) and tensile modulus (dashed line)**. Liu et al. (2010).

As a result, although increasing the extrusion temperature did not significantly influence the tensile properties (Figure 21B), both blending temperature and elastomer/ionomer ratio were found to play keys-roles in achieving super-toughness (great improvement of impact strength and strain at break) of the PLA-based ternary reactive blends (Figure 21A). This can attributed to the effective interfacial compatibilization at higher temperature (240°C).

**Figure 22:**
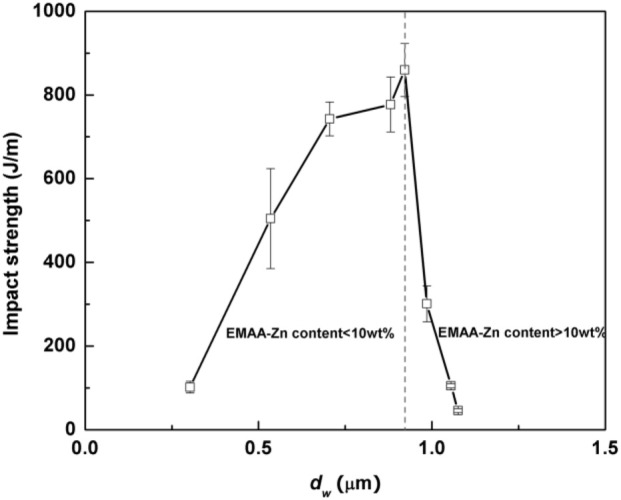
**Izod impact strength of PLA/EBA-GMA/EMAA-Zn (80/20-x/x) blends with total content of both modifiers fixed at 20 wt% as a function of weight-average particle diameter (dw)**. Adapted with permission from Liu et al. (2011).

The correlation between the particle size and impact toughness had revealed that there existed an optimum submicronic range of particle sizes of the dispersed domains for PLA super-toughening in this ternary blend system (Figure 22). Preliminary analysis of micromechanical deformation suggested that the high impact toughness observed for some ternary blends was attributed to the low cavitation resistance of the dispersed particles coupled with suitable interfacial adhesion. It was found that debonding mainly occurred around the relatively large particles together with fibrillated crazes and no cavitation when blended with an ethylene/n-butyl acrylate/glycidyl methacrylate terpolymer (EBA-GMA). Addition of a zinc ionomer of ethylene/methacrylic acid copolymer (EMAA-Zn) within the PLA/EBA-GMA blend gradually turned the morphology into a salami-like phase structure, which provides a low cavitation resistance coupled with suitable interfacial adhesion. Therefore, internal cavitation of the dispersed particles followed by the matrix shear yielding was predominant and resulted in the optimum impact strength. All of these examples regarding toughening mechanisms within PLA are not exhaustive but strengthens that toughness of PLA is a complex function which implies all of as-describe mechanisms (crazing, shear yielding, cavitation and debonding) and mode of fracture.

**Scheme 6:**
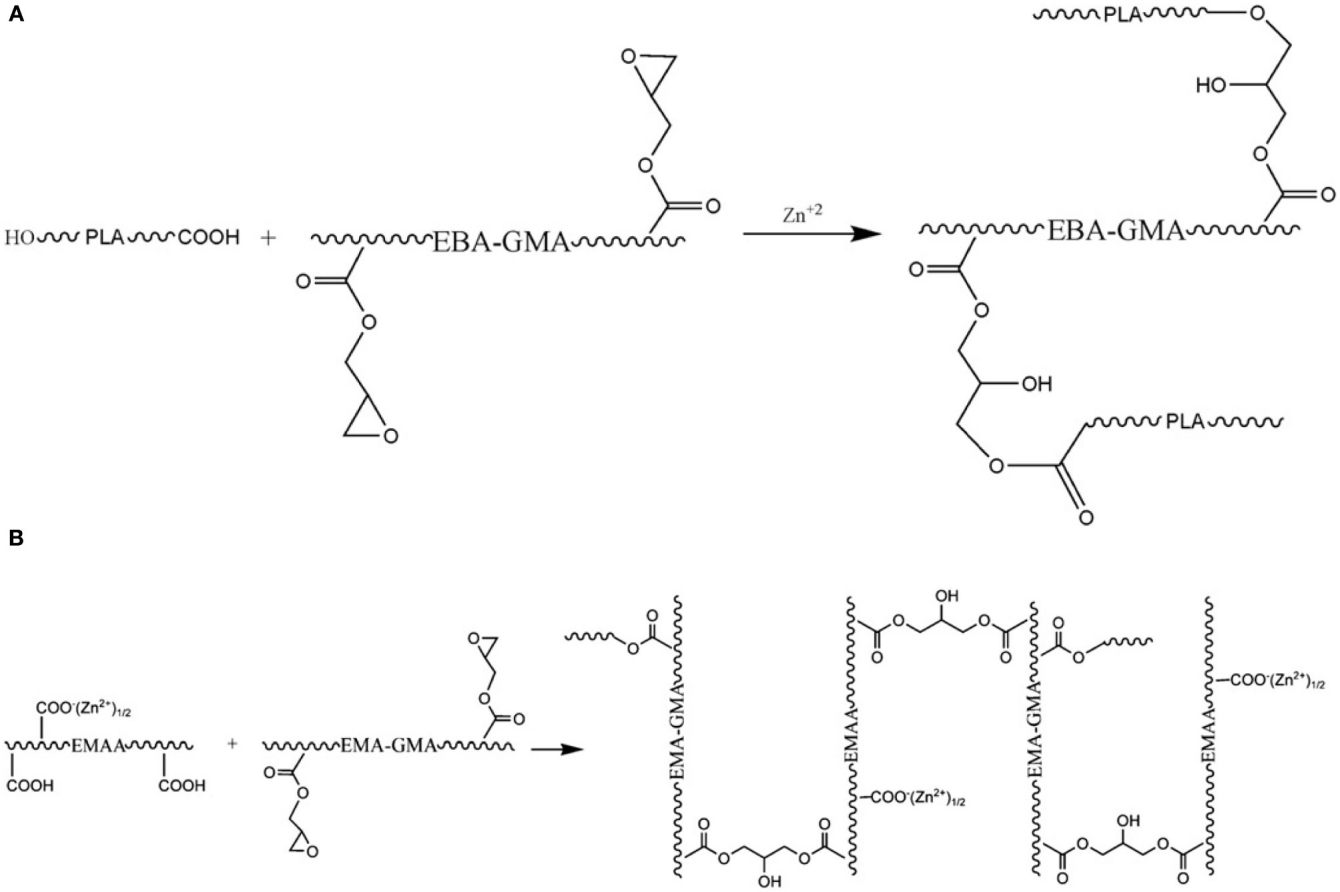
**(A)** Interfacial compatibilization reaction catalyzed by Zn2+ in the ionomer. **(B)** Schematic crosslinking reaction of EBA-GMA with ionomer during melt compounding [Reprinted from Song et al. (2012) with permission from Elseiver].

**Figure 23:**
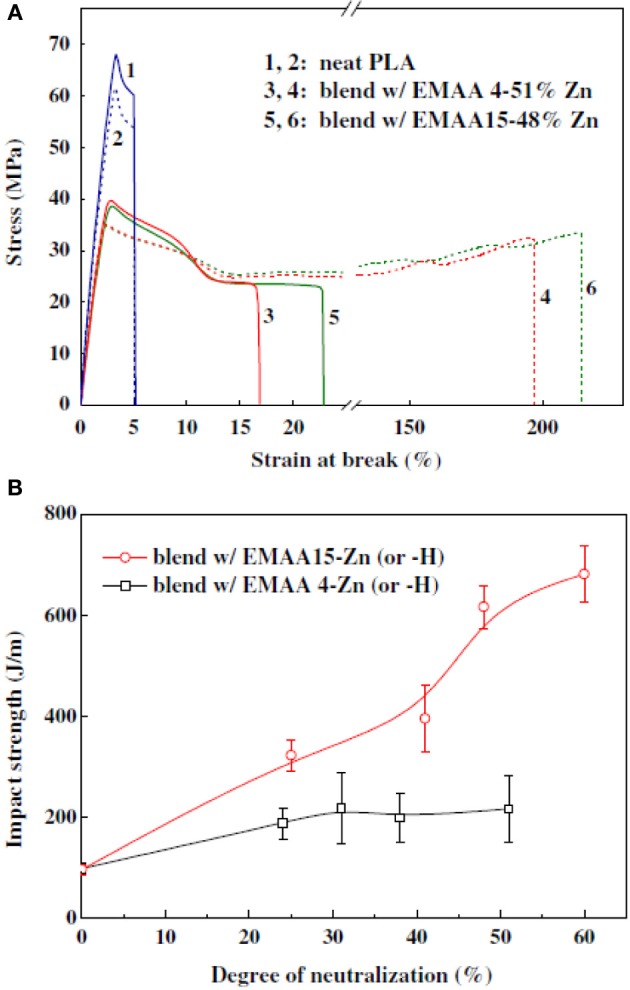
**(A)** Tensile stress–strain curves of neat PLA and PLA/EBA-GMA/EMAA-Zn (80/15/5, w/w) ternary blends under speed of extension of 2 inch/min (solid line) and 0.2 inch/min (dash line), respectively. **(B)** Effects of degree of neutralization and functionality of ionomers on the IS of PLA/EBA-GMA/EMAA-Zn (or EMAA-H) (80/15/5, w/w) blends [Reprinted from Song et al. (2012) with permission from Elseiver].

In a complementary study, Song et al. (2012) investigated the effect of the ionomer characteristics on reactions and properties of the PLA-based reactive ternary blends studied above (Schemes 6A,B). The ionomer was prepared by neutralizing the EMAA ionomer precursor with ZnO. It came out that the reactivity of the system and the interfacial compatibilization were drastically enhanced by increasing both the degree of neutralization (DN) of the ionomer and the methacrylic acid (MAA) content of ionomer precursor. As a result, the particle size and polydispersity of the dispersed phase reached the right optimum to greatly improve the impact toughness and tensile elongation at break of the material (Figures 23A,B).

#### Super-tough PLA alloy with greatly improved heat resistance

**Figure 24:**
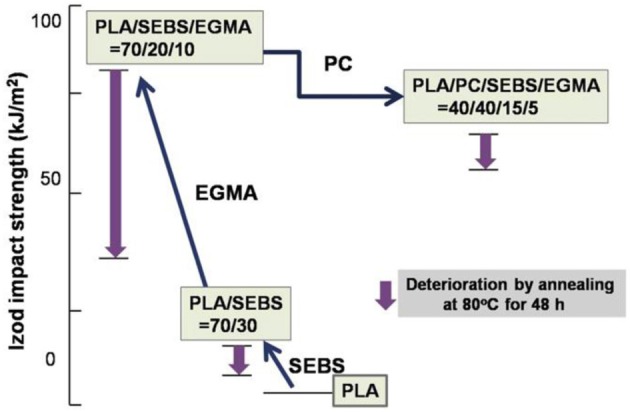
**Izod impact strength as a function of PLA-based material formulation** [Reprinted from Hashima et al. (2010) with permission from Elseiver].

Hashima et al. (2010) toughened PLA by blending it with hydrogenated styrene-butadiene-styrene block copolymer (SEBS) with the aid of reactive compatibilizer, poly(ethylene-*co*-glycidyl methacrylate) (EGMA). The high temperature property (HDT) and thermal ageing resistance were improved by further incorporating a ductile polymer with a high glass transition temperature, that is, polycarbonate (PC). Based on TEM, differential scanning calorimetry (DSC), and dynamic mechanical analysis (DMA), the author explained that the origin of the outstanding toughness and ageing resistance of the 4 component alloy; e.g., PLA/PC/SEBS/EGMA 40/40/5/5 (wt.% ratio), seems to come from the negative pressure effect of SEBS that dilates the plastic matrix consisting of PLA and PC to enhance the local segment motions. The phenomenon is briefly summarized in Figure 24.

**Figure 25:**
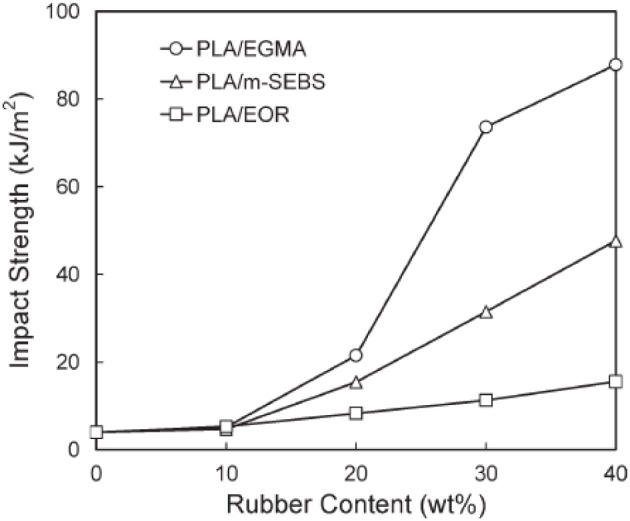
**Impact strength of PLA blends as function of rubber content**. Copyright (2012) Wiley; used with permission from Jiang et al. (2012).

Jiang et al. (2012) blended PLA with various commercial rubber components, i.e., poly (ethylene-glycidyl methacrylate) (EGMA), maleic anhydride grafted poly(styrene- ethylene/butylene-styrene) triblock elastomer (m-SEBS), and poly(ethylene-*co*-octene) (EOR) and compared their toughening effect on PLA (Figure 25). It was observed that: (i) EGMA was highly compatible due to its reaction with PLA, (ii) m-SEBS was less compatible with PLA, and (iii) EOR was incompatible with PLA. SEM and TEM revealed that a fine 3-D co-continuous microlayer structure was formed in the injection-moulded PLA/EGMA blends. This led to polymer blends with high toughness and very low linear thermal expansion both in the flow direction and in the transverse direction. The microlayer thickness of rubber in PLA blends was found to play key-roles in reducing the linear thermal expansion and achieving high toughness of the blends. Therefore, PLA blends with the notched impact strength over 20 times higher (ca. 90 kJ/m^2^) than that of the neat PLA (ca. 4 kJ/m^2^) were obtained by reactive blending of PLA and EGMA at 40 wt.% of rubber loading. It should be highlighted that the PLA/EGMA blend having both high impact resistance and low thermal expansion coefficient is of great importance in applications.

## Conclusion

In comparison with many other commodity thermoplastics, PLA presents many advantages, mainly its renewability, biodegradability, high stiffness and competitive cost production. The main problem for this biopolyester is its inherent brittleness due to a crazing deformation mechanism through which the polymer fails upon tensile and impact testing. Since many applications require high impact resistance and flexibility bio-based and/or biodegradable materials, several approaches aiming at toughening PLA has been investigated over the last decades. First of all, understanding the effect of the pristine microstructure modification on the mechanical performances was established. It has been demonstrated that de-aging and molecular orientation can improve the mechanical properties of PLA. However, such strategies require long and specific processes, which are not cost effective for an economical production of high performance PLA materials. Classically, compounding with softer polymers seems to be the best option for toughening PLA in costless way. The toughening effects of PLA blends are complicated as many parameters are concerned including the high interfacial adhesion between the matrix and the toughener, the domain size of the dispersed phase that should be ideally between 0.1 and 1.0 μm to improve the blend compatibility. The most common compatibilization way consists on the incorporation of block copolymers. Recently, chemical compatibilization *via* reactive extrusion has proven to be a very promising technology and more effective in improving the toughness of PLA blends. In some cases, outstanding toughness was successfully achieved, but accompanied with a compromise of the biodegradability and the initial stiffness of PLA. Therefore, the challenge pursues to develop a fully bio-based and biodegradable PLA-based material with a balance of outstanding mechanical properties.

### Conflict of interest statement

The authors declare that the research was conducted in the absence of any commercial or financial relationships that could be construed as a potential conflict of interest.
